# Contact guidance as a consequence of coupled morphological evolution and motility of adherent cells

**DOI:** 10.1007/s10237-022-01570-9

**Published:** 2022-04-27

**Authors:** Alberto Ippolito, Antonio DeSimone, Vikram S. Deshpande

**Affiliations:** 1 Department of Engineering, Cambridge University, Cambridge, CB2 1PZ UK; 2 SISSA – International School for Advanced Studies, Trieste, Italy; 3 The BioRobotics Institute, Scuola Superiore Sant’Anna, Pontedera, Pisa, Italy

**Keywords:** Contact guidance, Homeostasis, Motility, Fluctuations

## Abstract

Adherent cells seeded on substrates spread and evolve their morphology while simultaneously displaying motility. Phenomena such as contact guidance, viz. the alignment of cells on patterned substrates, are strongly linked to the coupling of morphological evolution with motility. Here, we employ a recently developed statistical thermodynamics framework for modelling the non-thermal fluctuating response of cells to probe this coupling. This thermodynamic framework is first extended via a Langevin style model to predict temporal responses of cells to unpatterned and patterned substrates. The Langevin model is then shown to not only predict the different experimentally observed temporal scales for morphological observables such as cell area and elongation but also the interplay of morphology with motility that ultimately leads to contact guidance.

**Supplementary Information:**

The online version contains supplementary material available at 10.1007/s10237-022-01570-9.

## Introduction

Living systems are characterised by a wide variety of timescales governing key biological processes at different length scales. For example, on a macroscopic scale, it is well known that animal species have their own circadian rhythm (Aschoff 1979; Ohata et al. 1999) that governs their daily behaviours, such as feeding and resting. On a microscopic scale, cells follow a cell cycle, which is a sequence of cellular phases such growth and division (Johnson and Walker 1999; Kastan and Burtek 2004). These timescales are critical for cell development and are controlled by complex molecular pathways (Johnson and Walker 1999; Kastan and Burtek 2004; Stanewsky et al. 1998; Kume et al. 1999). Another example pertains to the in vitro spreading of adherent single cells on substrates. Experiments have demonstrated that the spreading and elongation rates differ in time by an order of magnitude (Kesavan et al. 2014; Nisenholz et al. 2014). The rates of spreading and elongation were also observed to be non-constant, which suggested that distinct temporal phases may exist in a cell’s morphological evolution.

Another important timescale pertaining to in vitro experiments of adherent cells is associated with cell motility. Motility occurs via large and co-ordinated morphological changes in the cell, e.g. the treadmilling mechanism in adherent cells, whereby cells move by tugging on the substrate by exploiting focal adhesions (Balaban et al. 2001; Bell et al. 1984). Therefore, the motility and morphological change timescales are expected to be interlinked. It is well known that substrate properties such as stiffness (Hadden et al. 2017; Ulrich et al. 2009), chemical composition (Engler et al. 2004; Carter 1965, 1967) and topology (Buskermolen et al. 2020; Chang et al. 2013; Ray et al. 2017) strongly affect single-cell morphological and motile behaviour. Experiments have shown that changing the substrate stiffness alters both the spreading timescales of adherent cells and the speed at which they explore the environment (Asano et al. 2017; Ghibaudo et al. 2008; Pelham and Wang 1997). Guidance provided by substrate anisotropy, i.e. contact guidance (Guido and Tranquillo 1993; Dunn and Heath 1976; Weiss 1945), also influences cell spreading and motile behaviour. In fact, cells elongate more and faster on adhesive channels (Huang and Donald 2015) and this is also accompanied by an enhanced alignment of the biochemical force generating machinery within the cell, viz. stress-fibres (Buskermolen et al. 2019). Measurements have also reported a faster directional exploration speed in confined settings (Pathak and Kumar 2012). However, physical insights into the interplay between morphological changes and motility for cells on anisotropic substrates are lacking despite their importance for understanding cell guidance.

**Fig. 1:**
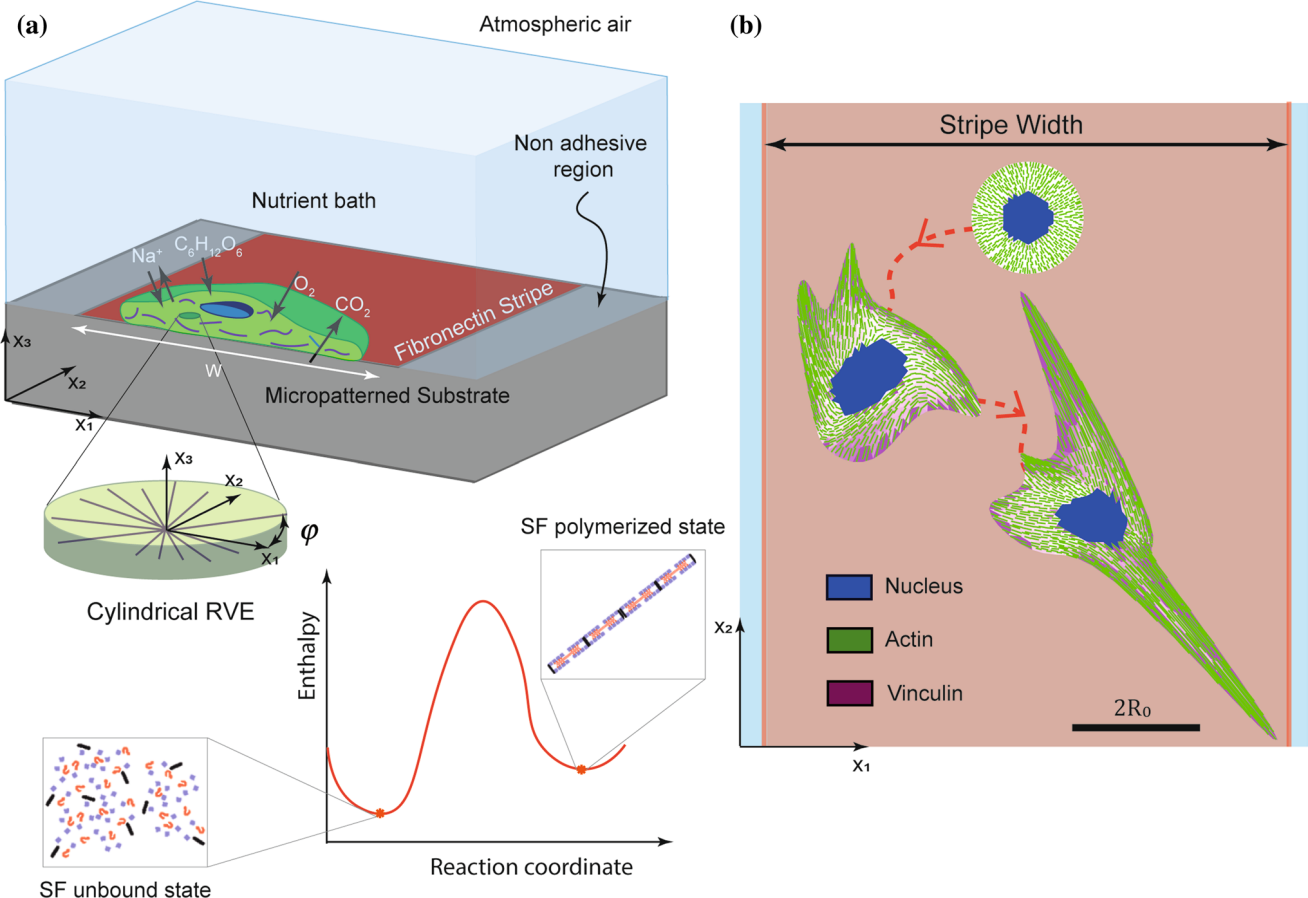
Simulation set-up. **a** Single cell placed on a fibronectin stripe of fixed width $W$. The cell exchanges high-energy nutrients with the nutrient bath. The components of the cell modelled explicitly include an elastic nucleus and cytoplasm as well as the contractile stress-fibres in their polymerised state in equilibrium with their unbound components within the cytoplasm. **b** Temporal evolution of an adherent cell on a fibronectin stripe. In the simulation, cells are modelled as two-dimensional bodies in the ${x}_{1}-{x}_{2}$ plane lying on a substrate. All cells are seeded from their state in suspension and then spread and change shape while simultaneously exploring the substrate. The images in (**b**) are representative images from simulations with the scalebar of $2{R}_{0}$ the diameter of the circular cell in its elastic resting state

The aim of this theoretical work is to understand the timescales governing phenomena, such as contact guidance, that emerge due to the coupling of morphological evolution with cell motility. We propose a novel framework that we call the Homeostatic Langevin Equation (HLE) that is an extension to the Homeostatic ensemble (Shishvan et al. 2018) developed to understand the long timescale or stationary responses of cells. The HLE recognises the non-thermal fluctuating response of the cells and is used to simulate the temporal evolution of isolated cells seeded on unpatterned and patterned substrates. A schematic of the simulation set-up for a cell seeded on a substrate with a fibronectin stripe of width $W$ is shown in Fig. 1a along with a representative prediction in Fig. 1b. The cell seeded from suspension is circular, and the HLE will predict its coupled morphological evolution along with its motility as shown in Fig. 1b. Key experimental observables such as cell area, cell shape, distribution of cytoskeletal proteins such as stress-fibre distributions, shape of nucleus and focal adhesion distributions are outcomes of the simulations. These predictions are used to extract the timescales of morphological evolution and cell motility and thereby develop an understanding of the mechanisms and processes that result in the guidance of cells on patterned substrates.

## Modelling formulation

The interaction of cells with environmental cues (e.g. mechanical, chemical) has a profound influence on cell response. For example, not only do morphological observables such as cell area and aspect ratio increase with increasing substrate stiffness (Kesavan et al. 2014; Nisenholz et al. 2014) but cell motility also exhibits a biphasic behaviour on stiffer substrates (Peyton and Putnam 2005; Pathak and Kumar 2011). While different modelling frameworks are used to understand the influence of environmental cues on cell morphology (Vigliotti et al. 2015; Shenoy et al. 2016; Bengassser et al. 2013) and cell motility (Stokes et al. 1991; Klank et al. 2017), the interplay of the underlying mechanisms remains elusive. Our aim here is to develop a unified framework with the objective of better elucidating the underlying mechanisms. We do this by extending the recently developed *homeostatic mechanics framework* (Shishvan et al. 2018), which predicts the distribution of morphological states that an adherent cell assumes during the interphase period of the cell cycle. The mentioned framework lacks temporal information, and here, we extend the formulation to enable predictions of the temporal evolution of morphological observables alongside cell motility as a function of environmental cues.

### A brief overview of the homeostatic mechanics framework

The homeostatic mechanics framework recognises that a cell is an open system which exchanges nutrients with the surrounding nutrient bath (Fig. 1a); see Shishvan et al. (2018) for further details. These high-energy nutrient exchanges fuel large fluctuations (much larger than thermal fluctuations) in the cell response associated with various intracellular biochemical processes. The cell uses these biochemical processes to maintain itself in the homeostatic state.

Specifically, homeostasis is the ability of a living cell to remain out of thermodynamic equilibrium by maintaining its various molecular species at a specific average number that is independent of the environment (Gordon 2013). This average number is sustained over all the non-thermal fluctuations of the cell (at-least over the interphase period of the cell cycle and in the absence of any imposed shock such as starving the cell of nutrients). The implication is that over the fluctuations of the cell from any reference state, $\langle \Delta {N}_{i}\rangle =0$ where $\Delta {N}_{i}$ is the change in the number of molecules of species $i$ from its reference value with $\langle \mathrm{x}\rangle$ denoting the average of $\mathrm{x}$ over the ensemble of states sampled over the non-thermal fluctuations. These fluctuations alter the cell morphology, and each morphological microstate (cell shape, protein distribution etc.) has an equilibrium Gibbs free energy $G={\sum }_{i}{\mu }_{i}{N}_{i}$, where ${\mu }_{i}$ is the chemical potential of species $i$. Using the Gibbs–Duhem relation, we then rewrite this in terms of the reference state as $G={G}_{\mathrm{ref}}+{\sum }_{i}{\mu }_{i}^{0}{\Delta N}_{i}$, where now ${\mu }_{i}^{0}$ is the chemical potential of species $i$ in the reference state and ${G}_{\mathrm{ref}}$ is the equilibrium Gibbs free energy of the cell in its reference state. Upon employing the homeostatic constraint that $\langle \Delta {N}_{i}\rangle =0$, we have $\langle G\rangle ={G}_{\mathrm{ref}}$, i.e. irrespective of the environment, the ensemble average Gibbs free energy is constant. This is a universal constraint that quantifies the fact that living cells maintain themselves out of thermodynamic equilibrium but yet attain a stationary state which is the homeostatic state. While the above constraint specifies the average state of the cell, it remains to determine the distribution of states the cell assumes that satisfies this average. As mentioned, cells do not remain in a single microstate but fluctuate between different configurations after seeding. Cells explore these different morphological microstates thanks to several biochemical processes, such as actin polymerisation and treadmilling. The objective of the homeostatic mechanics framework is to capture the different configurations by adopting the *ansatz* that the observed distribution of cell shapes is the one with the overwhelming number of microstates, i.e. the distribution that maximises the *morphological entropy* subject to the homeostatic constraint, i.e. $\langle G\rangle ={G}_{\mathrm{ref}}={G}_{\mathrm{S}}$, with ${G}_{\mathrm{S}}$ being the free energy of a cell in suspension, and to any other geometrical constraints, such as confinement, imposed by adhesive patterns on substrates. The distribution of states is denoted as an equilibrium distribution, where “equilibrium” means in a homeostatic state.

**Fig. 2:**
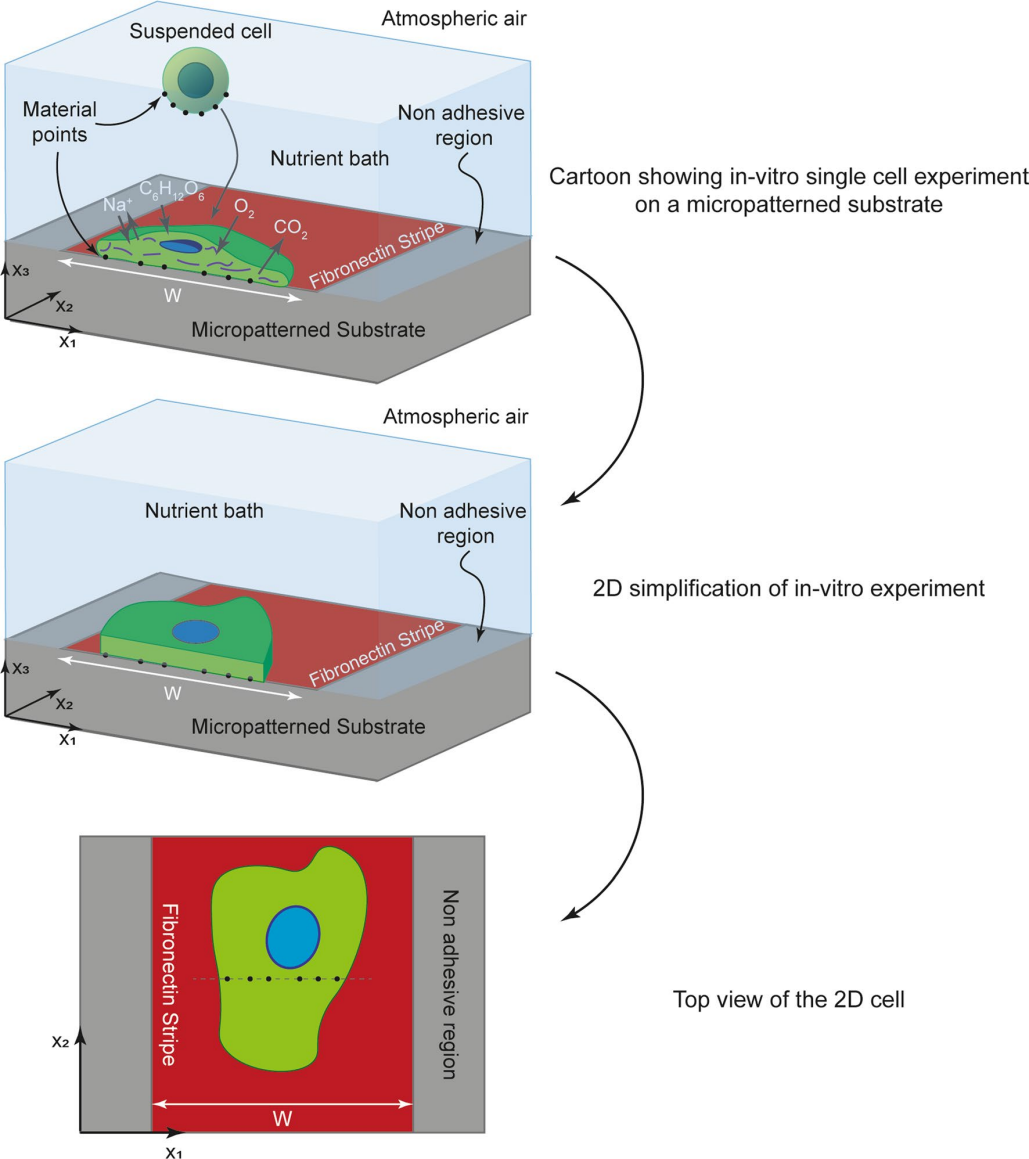
Simplification of an in vitro cell to a 2-dimensional body lying on the ${x}_{1}-{x}_{2}$ plane

In this study, cells are modelled as two-dimensional bodies in the $x_{1} - x_{2}$ plane lying on a substrate (Fig. 2). In this two-dimensional context, the cell morphology is defined by the positional vectors ${}_{{}}^{\left( q \right)} r_{i}$ $\left( {i = 1,2 } \right)$ of the $q = 1, \ldots ,M$ control points used to specify the cell shape. Denoting the positions of the control points in morphological microstate $\left( j \right)$ as $\tilde{r}^{\left( j \right)} = \left\{ {{}_{{}}^{\left( 1 \right)} r_{i}^{\left( j \right)} , \ldots ,{ }{}_{{}}^{\left( M \right)} r_{i}^{\left( j \right)} } \right\}$, the Gibbs free energy of the cell in microstate $\left( j \right)$ is $G^{\left( j \right)} \equiv G\left( {\tilde{r}^{\left( j \right)} } \right)$. Shishvan et al. (2018) demonstrated that the cell in homeostasis assumes a given morphological microstate $\left( j \right)$ with probability

1 $$P_{eq}^{(j)} \equiv P_{eq} \left( {\widetilde{r}^{\left( j \right)} } \right) = \frac{1}{Z}\exp \left( { - \zeta G^{\left( j \right)} } \right),$$

where $Z\equiv {\sum }_{j}\mathrm{exp}(-\zeta {G}^{\left(j\right)})$ is the partition function of the morphological microstates, and the distribution parameter $\zeta$ emerges from the homeostatic constraint $\langle {G}^{(j)}\rangle \equiv {\sum }_{j}{{P}_{\mathrm{eq}}^{\left(j\right)}G}^{\left(j\right)}={G}_{\mathrm{S}}$. Thus, 1/$\zeta$ in (1) is referred to as the *homeostatic temperature,* and it sets the equilibrium distribution of morphological microstates of the cell (also referred to as the *homeostatic ensemble*) as an analogous quantity to the thermodynamic temperature of the canonical ensemble (Gibbs 1902).

The 2D cell in microstate $(j)$ is described by the $q=1,\dots ,M$ positional vectors ${}_{{}}^{\left( q \right)} r_{i}$ $(i=\mathrm{1,2})$. Here, we describe the numerical scheme used to define these vectors. In the 2D context of cells on substrates, describing a morphological microstate reduces to specifying the position of all material points where the cell is in contact with the substrate. Thus, given the location of material points ${X}_{i}$ on the elastically undeformed configuration, we impose a displacement field ${u}_{i}^{\left(j\right)}({X}_{i})$ to obtain the displaced coordinates of these points ${x}_{i}^{\left(j\right)}={X}_{i}+{u}_{i}^{\left(j\right)}$ which defines the morphological microstate $\left(j\right)$. The definition of the morphological microstate is then completed in the 2D setting by assuming that these points on the cell are connected to material points on the substrate in the same location ${x}_{i}^{\left(j\right)}$.

The cell is modelled as a continuum, and thus, $u_{i}^{\left( j \right)}$ is a continuous field. We define $u_{i}^{\left( j \right)}$ via Non-Uniform Rational B-Splines (NURBS) such that the morphological microstate is defined by $M$ control points of coordinates $\left[ {{}_{{}}^{\left( q \right)} r_{1} ,{}_{{}}^{\left( q \right)} r_{2} } \right]$. In all the numerical results presented here, we employ $M = 16$ with $4 \times 4$ control points ${}_{{}}^{\left( q \right)} r_{1}$ and ${}_{{}}^{\left( q \right)} r_{2}$ governing the displacements in the $x_{1}$ and $x_{2}$ directions, respectively. We construct the NURBS by using third-degree polynomials in both the ${x}_{1}$ and ${x}_{2}$ directions and by choosing two knots both with multiplicity four located at the extrema of the interval. The choice of these parameters enforces restriction on the sampled morphological microstates, i.e. features with wavelengths below the characteristic wavelength of the NURBS are not captured and thus typically this wavelength is chosen to represent the minimum width of a filopodium expected for the selected cell type (Shishvan et al. 2018; Buskermolen et al. 2019). Given the displacement field${u}_{i}^{\left(j\right)}$, we evaluate the Gibbs free energy ${G}^{(j)}$ using model described below. The cell is discretised by constant strain triangles of size$e\approx {R}_{0}/10$, where ${R}_{0}$ is the radius of the cell in its elastic resting state.

The numerical methods used for sampling via Markov chain Monte Carlo (MCMC) the morphological microstates and to obtain 1/$\zeta$ are the same as Shishvan et al. (2018) and Buskermolen et al. (2019), and a brief description is provided in Appendix A.

### Gibbs free energy of a morphological microstate

The implementation of the homeostatic mechanics approach described above requires a specific model for the Gibbs free energy of the cell-substrate system in a given morphological state. Modelling all the elements of the cell is unrealistic, given that many of their kinetics are still unknown, and often not required, as specific components are known to determine and control the cell response to different environmental cues. Here, we are interested in investigating the response of cells to adhesive patterning of the substrates. These cues are known to guide single-cell behaviour resulting in significant cell alignment bias as well as remodelling of the stress-fibre cytoskeleton. Thus, we use a model that includes stress-fibre cytoskeleton introduced by Vigliotti et al. (2016) and subsequently modified in Shishvan et al. (2018) as well as passive elasticity of the cytoplasm and nucleus.

The Gibbs free energy of a morphological microstate $(j)$ of the system comprises contributions from the cell and the substrate and is written as

2 $${G}^{\left(j\right)}={G}_{\mathrm{cell}}^{(j)}+{G}_{\mathrm{sub}}^{(j)}.$$

For the sake of brevity, we drop the superscript $(j)$ throughout this section with the understanding that we are always discussing a given morphological microstate. The system (i.e. cell plus substrate) is within a nutrient bath and under atmospheric pressure as illustrated in Fig. 2. Then, taking atmospheric pressure as the reference zero state, $G$ is given by

3 $$G={F}_{\mathrm{cell}}+{F}_{\mathrm{sub}}-{\int }_{{S}_{T}}{T}_{i}^{e}{u}_{i}dS={F}_{\mathrm{cell}}+{F}_{\mathrm{sub}},$$

as ${T}_{i}^{e}=0$ on the surface ${S}_{T}$ of the system exposed to the environment with ${F}_{\mathrm{cell}}$ and ${F}_{\mathrm{sub}}$ the corresponding Helmholtz free energies of the cell and substrate, respectively. For the linear substrate model, the substrate Helmholtz free energy ${F}_{\mathrm{sub}}=-{G}_{\mathrm{sub}}$. The free energy of the cell comprises four contributions such that

4 $${F}_{\mathrm{cell}}={\int }_{{V}_{\mathrm{cell}}}\left({f}_{\mathrm{cyto}}+{f}_{\mathrm{ec}}+{f}_{\mathrm{passive}}\right)dV+{\int }_{{S}_{I}}{f}_{\mathrm{adh}}dS,$$

where ${f}_{\mathrm{cyto}},{f}_{\mathrm{ec}},{f}_{\mathrm{passive}}$ are the cytoskeletal, energy carrier and passive Helmholtz free energies per unit volume in the cell, while ${f}_{\mathrm{adh}}$ is the adhesive protein Helmholtz free energy per unit area over the interface ${S}_{I}$ between the cell and substrate. We define

5 $${F}_{\mathrm{active}}={\int }_{{V}_{\mathrm{cell}}}\left({f}_{\mathrm{cyto}}+{f}_{\mathrm{ec}}\right)dV+{\int }_{{S}_{I}}{f}_{\mathrm{adh}}dS,$$

and the corresponding Gibbs free energy of the active components is given by the Legendre transform as

6 $$G_{{active}} = \mathop \smallint \limits_{{V_{{cell}} }} \left( {g_{{cyto}} + g_{{ec}} } \right)dV + \mathop \smallint \limits_{{S_{I} }} g_{{adh}} dS = F_{{active}} - \mathop \smallint \limits_{{V_{{cell}} }} \sigma _{{ij}}^{A} \varepsilon _{{ij}} dV - \mathop \smallint \limits_{{S_{I} }} T_{i} \Delta _{i} dS,$$

where the active stress ${\sigma }_{ij}^{A}$ is related to the strain ${\varepsilon }_{ij}$ via ${\sigma }_{ij}^{A}\equiv \partial ({f}_{\mathrm{cyto}}+{f}_{\mathrm{ec}})/\partial {\varepsilon }_{ij}$, while the surface tractions ${T}_{i}$ and stretch ${\Delta }_{i}$ of the adhesion proteins are related by ${T}_{i}\equiv \partial {f}_{\mathrm{adh}}/\partial {\Delta }_{i}$. The free energy associated with the energy carriers drives mechanical work and so we assume

7 $${\int }_{{V}_{\mathrm{cell}}}{g}_{\mathrm{ec}}dV=-{\int }_{{V}_{\mathrm{cell}}}{\sigma }_{ij}^{A}{\varepsilon }_{ij}dV-{\int }_{{S}_{I}}{T}_{i}{\Delta }_{i}dS,$$

and substituting (7) into (6) gives

8 $${F}_{\mathrm{active}}={\int }_{{V}_{\mathrm{cell}}}{g}_{\mathrm{cyto}}dV+{\int }_{{S}_{I}}{g}_{\mathrm{adh}}dS.$$

The Gibbs free energy of the system is then as follows:

9 $$G={F}_{\mathrm{cell}}+{F}_{\mathrm{sub}}={\int }_{{V}_{\mathrm{cell}}}{(g}_{\mathrm{cyto}}+{f}_{\mathrm{passive}})dV+{\int }_{{S}_{I}}{g}_{\mathrm{adh}}dS+ {F}_{\mathrm{sub}}.$$

We assume that the collagen coating of both the unpatterned and patterned substrates is dense, and thus, there is not any impedance in the formation of focal adhesions; the term ${\int }_{{S}_{I}}{g}_{\mathrm{adh}}dS$ is assumed constant for ever configuration. Additionally, the underlying substrate is assumed to be rigid, i.e. ${F}_{\mathrm{sub}}=0$. We proceed to discuss the formulation for ${g}_{\mathrm{cyto}}$ and ${f}_{\mathrm{passive}}$.

#### Calculation of cell free energy

Calculation of ${g}_{\mathrm{cyto}}$ and ${f}_{\mathrm{passive}}$ involves modelling three elements within the cell: (i) a passive elastic contribution from elements such as the cell membrane, intermediate filaments, and microtubules in the cytoplasm, (ii) an active contribution from the contractile actomyosin stress-fibres that are modelled explicitly and (iii) the nucleus modelled as a passive elastic body. These are modelled using the framework of Vigliotti et al. (2016) and the subsequent modifications of Shishvan et al. (2018) and Buskermolen et al. (2019). We shall first describe the modelling of the active actomyosin stress-fibres in the cytoplasm and then discuss the elastic model of both the nucleus and the cytoplasm.

Consider an incompressible two-dimensional (2D) cell (both the cytoplasm and nucleus are assumed to be incompressible) of thickness ${b}_{0}$, radius ${R}_{0}$ and volume ${V}_{0}$ in its elastic resting state comprising a nucleus of volume ${V}_{\mathrm{N}}$ and cytoplasm of volume ${V}_{\mathrm{C}}$ such that ${V}_{0}={V}_{\mathrm{N}}+{V}_{\mathrm{C}}$ (Fig. 1a). We assume a cylindrical representative element (RVE) of volume ${V}_{\mathrm{R}}=\pi {b}_{0}{\left({n}^{\mathrm{R}}{\mathcal{l}}_{0}/2\right)}^{2}$, where ${\mathcal{l}}_{0}$ is the length of a stress-fibre functional unit in its ground state and ${n}^{\mathrm{R}}$ is the number of these ground-state functional units within the cytoplasm. The average number of functional units (polymerised and unbound) in the RVE is then ${N}_{0}={N}_{0}^{\mathrm{T}}{V}_{\mathrm{R}}/{V}_{\mathrm{C}}$, where ${N}_{0}^{\mathrm{T}}$ is the total number of functional units present in the cell. The state of the stress-fibres in the RVE located in ${x}_{i}$ is described by their angular concentration $\eta \left({x}_{i},\phi \right)$ and the number of functional units $n({x}_{i},\phi )$ in series along the length of each stress-fibre. The angle $\phi$ is measured in the undeformed configuration and measures the orientation of the stress-fibre bundle with respect to the ${x}_{2}-$ direction of the stripe (Fig. 1a). At steady state, Vigliotti et al. (2016) showed that the number ${n}^{\mathrm{ss}}$ of functional units within the stress-fibres is

10 $${\widehat{n}}^{\mathrm{ss}}\equiv \frac{{n}^{\mathrm{ss}}}{{n}^{\mathrm{R}}}=\frac{\lambda ({x}_{i},\phi )}{1+{\stackrel{\sim }{\varepsilon }}_{\mathrm{nom}}^{\mathrm{ss}}} ,$$

where ${\stackrel{\sim }{\varepsilon }}_{\mathrm{nom}}^{\mathrm{ss}}$ is the strain at steady state within a functional unit of the stress-fibres, and $\lambda ({x}_{i},\phi )$ is the cell stretch in direction $\phi$, i.e. component of the left stretch tensor in direction $\phi$. The chemical potential of the functional units within the stress-fibres is given in terms of the Boltzmann constant ${k}_{\mathrm{B}}$ and of the normalised number ${\widehat{N}}_{\mathrm{L}}$ of lattice sites available to unbound proteins by

11 $${\chi }_{\mathrm{b}}=\frac{{\mu }_{\mathrm{b}}}{{n}^{\mathrm{R}}}+{k}_{\mathrm{B}}T \mathrm{ln}\left[{\left(\frac{\pi \widehat{\eta } {\widehat{n}}^{\mathrm{ss}}}{{\widehat{N}}_{\mathrm{u}}\left(1-\frac{\widehat{\eta }}{{\widehat{\eta }}_{\mathrm{max}}}\right)}\right)}^{\frac{1}{{n}^{\mathrm{ss}}}}\left(\frac{{\widehat{N}}_{\mathrm{u}}}{\pi {\widehat{N}}_{\mathrm{L}}}\right)\right] ,$$

where ${\widehat{N}}_{\mathrm{u}}\equiv {N}_{\mathrm{u}}/{N}_{0}$ is the normalised concentration of the unbound stress-fibre proteins in terms of the concentration ${N}_{\mathrm{u}}$ of the unbound, $\widehat{\eta }\equiv \eta {n}^{\mathrm{R}}/{N}_{0}$ and ${\widehat{\eta }}_{\mathrm{max}}$ is the maximum normalised value of $\widehat{\eta }$ corresponding to full occupancy of all available sites for stress-fibres (in a specific direction). Here, the enthalpy ${\mu }_{\mathrm{b}}$ of ${n}^{\mathrm{R}}$ bound functional units at steady state is given in terms of the isometric stress-fibre stress ${\sigma }_{\mathrm{max}}$ and the internal energy ${\mu }_{\mathrm{b}0}$ as

12 $${\mu }_{\mathrm{b}}={\mu }_{\mathrm{b}0}-{\sigma }_{\mathrm{max}}\Omega \left(1+{\stackrel{\sim }{\varepsilon }}_{\mathrm{nom}}^{\mathrm{ss}}\right),$$

where $\Omega$ is the volume of ${n}^{\mathrm{R}}$ functional units. By contrast, the chemical potential of the unbound proteins is independent of stress and given in terms of the internal energy ${\mu }_{\mathrm{u}}$ of the unbound proteins as

13 $${\chi }_{\mathrm{u}}=\frac{{\mu }_{\mathrm{u}}}{{n}^{\mathrm{R}}}+{k}_{\mathrm{B}}T \mathrm{ln}\left(\frac{{\widehat{N}}_{\mathrm{u}}}{\pi {\widehat{N}}_{\mathrm{L}}}\right) .$$

For a fixed configuration of the 2D cell (i.e. a fixed stretch distribution $\lambda ({x}_{i},\phi )$), the contribution to the specific Gibbs free energy of the cell from the stress-fibres then follows as

14 $${g}_{\mathrm{cyto}}={\rho }_{0}\left({{\widehat{N}}_{\mathrm{u}}\chi }_{\mathrm{u}}+{\int }_{-\pi /2}^{\pi /2}\widehat{\eta } {\widehat{n}}^{\mathrm{ss}}{\chi }_{\mathrm{b}}d\varphi \right) ,$$

where ${\rho }_{0}\equiv {N}_{0}/{V}_{\mathrm{R}}$ is the number of protein packets per unit reference volume available to form functional units in the cell. However, we cannot yet evaluate ${g}_{\mathrm{cyto}}$ as ${\widehat{N}}_{\mathrm{u}}({x}_{i})$ and $\widehat{\eta }({x}_{i},\phi )$ are unknown and their value will follow from the chemical equilibrium of the cell as we shall discuss subsequently.

The total stress ${\Sigma }_{ij}$ within the cell includes contributions from the passive elasticity provided mainly by the intermediate filaments of the cytoskeleton attached to the nuclear and plasma membranes and the microtubules, as well as the active contractile stresses of the stress-fibres. The total Cauchy stress is written by an additive decomposition as

15 $${\Sigma }_{ij}={\sigma }_{ij}^{A}+{\sigma }_{ij}^{\mathrm{p}} ,$$

where ${\sigma }_{ij}^{A}$ and ${\sigma }_{ij}^{\mathrm{p}}$ are the active and passive Cauchy stresses, respectively. In the 2D setting with the cell lying in the ${x}_{1}-{x}_{2}$ plane, the active stress is given in terms of the volume fraction ${\mathcal{H}}_{0}$ of the stress-fibre proteins as

16 $$\left[\begin{array}{cc}{\sigma }_{11}^{A}& {\sigma }_{12}^{A}\\ {\sigma }_{12}^{A}& {\sigma }_{22}^{A}\end{array}\right]=\frac{{\mathcal{H}}_{0}{\sigma }_{\mathrm{max}}}{2}\underset{-\pi /2}{\overset{\pi /2}{\int }}\widehat{\eta }\lambda (\phi )\left[\begin{array}{cc}{2\mathrm{sin}}^{2}\phi & -\mathrm{sin} 2\phi \\ -\mathrm{sin} 2\phi & {2\mathrm{cos}}^{2}\phi \end{array}\right]d\phi ,$$

where $\phi$ is the angle of the stress-fibre measured with respect to ${x}_{2}$, in the current deformed configuration. The passive elasticity in the 2D setting is given by a 2D specialisation of the Ogden (1972) hyperelastic model as derived in (Shishvan et al. 2018). The strain energy density function of this 2D Ogden model is

17 $${\Psi }_{\mathrm{C}}\equiv \frac{2{\mu }_{\mathrm{C}}}{{m}_{\mathrm{C}}^{2}}\left[{\left(\frac{{\lambda }_{\mathrm{I}}}{{\lambda }_{\mathrm{II}}}\right)}^{\frac{{m}_{\mathrm{C}}}{2}}+{\left(\frac{{\lambda }_{\mathrm{II}}}{{\lambda }_{\mathrm{I}}}\right)}^{\frac{{m}_{\mathrm{C}}}{2}}-2\right]+\frac{{\kappa }_{\mathrm{C}}}{2}{\left({\lambda }_{\mathrm{I}}{\lambda }_{\mathrm{II}}-1\right)}^{2}+\mathcal{P},$$

for the cytoplasm and

18 $${\Psi }_{\mathrm{N}}\equiv \frac{2{\mu }_{\mathrm{N}}}{{m}_{\mathrm{N}}^{2}}\left[{\left(\frac{{\lambda }_{\mathrm{I}}}{{\lambda }_{\mathrm{II}}}\right)}^{\frac{{m}_{\mathrm{N}}}{2}}+{\left(\frac{{\lambda }_{\mathrm{II}}}{{\lambda }_{\mathrm{I}}}\right)}^{\frac{{m}_{\mathrm{N}}}{2}}-2\right]+\frac{{\kappa }_{\mathrm{N}}}{2}{\left({\lambda }_{\mathrm{I}}{\lambda }_{\mathrm{II}}-1\right)}^{2}+\mathcal{P},$$

for the nucleus where ${\lambda }_{\mathrm{I}}$ and ${\lambda }_{\mathrm{II}}$ are the principal stretches, ${\mu }_{\mathrm{C}}$ (${\mu }_{\mathrm{N}}$) and ${\kappa }_{\mathrm{C}}$ (${\kappa }_{\mathrm{N}}$) the shear modulus and in-plane bulk modulus of cytoplasm (nucleus), respectively, while ${m}_{\mathrm{C}}$ (${m}_{\mathrm{N}}$) is a material constant governing the nonlinearity of the deviatoric elastic response of cytoplasm (nucleus). The term $\mathcal{P}$ is a penalty term introduced to include an elastic penalty that prevents significant compression of the cytoplasm/nucleus in the ${x}_{1}-{x}_{2}$ plane and given by $\mathcal{P}\equiv \overline{\kappa }\mathcal{H}\left({J}_{\mathrm{c}}-{\lambda }_{\mathrm{I}}{\lambda }_{\mathrm{II}}\right){\left({\lambda }_{\mathrm{I}}{\lambda }_{\mathrm{II}}-{J}_{\mathrm{c}}\right)}^{2}$, where $\mathcal{H}\left(\bullet \right)$ is the Heaviside step function, ${J}_{c}$ a non-dimensional constant that sets when this term becomes nonzero and $\overline{\kappa }$ sets the magnitude of the penalty.

The cell is assumed to be incompressible, and thus throughout the cell, we set the principal stretch in the ${x}_{3}-$ direction${\lambda }_{\mathrm{III}}=1/({\lambda }_{\mathrm{I}}{\lambda }_{\mathrm{II}})$. The passive Cauchy stress then follows as ${\sigma }_{ij}^{\mathrm{p}}{p}_{j}^{(k)}={\sigma }_{k}^{\mathrm{p}}{p}_{i}^{(k)}$ in terms of the principal passive Cauchy stresses ${\sigma }_{k}^{\mathrm{p}}$ ($\equiv {\lambda }_{k}\partial {\Psi }_{\mathrm{C}}/\partial {\lambda }_{k}$ for the cytoplasm and $\equiv {\lambda }_{k}\partial {\Psi }_{\mathrm{N}}/\partial {\lambda }_{k}$ for the nucleus) and the unit vectors ${p}_{j}^{(k)} \left(k=\mathrm{I},\mathrm{ II}\right)$ denoting the principal directions $.$ The passive Helmholtz free energy of the cell is then ${f}_{\mathrm{passive}}={\Psi }_{\mathrm{C}}$ in the cytoplasm and ${f}_{\mathrm{passive}}={\Psi }_{\mathrm{N}}$ in the nucleus.

The equilibrium of a morphological microstate reduces to two conditions (Shishvan et al 2018): (i) mechanical equilibrium with ${\Sigma }_{ij,j}=0$ throughout the system, and (ii) intracellular chemical equilibrium. This second condition implies that the chemical potentials of bound and unbound stress-fibre proteins are equal throughout the cell (${\chi }_{\mathrm{u}}\left({x}_{i}\right)={\chi }_{\mathrm{b}}\left({x}_{i},\phi \right)=\mathrm{constant}$) which constrains $\widehat{\eta }({x}_{i},\phi )$ and ${\widehat{N}}_{\mathrm{u}}$, by combining (11) and (13), as

19 $$\widehat{\eta }\left({x}_{i},\phi \right)=\frac{{\widehat{N}}_{\mathrm{u}} {\widehat{\eta }}_{\mathrm{max}}\mathrm{exp}\left[\frac{{\widehat{n}}^{\mathrm{ss}}({\mu }_{\mathrm{u}}-{\mu }_{\mathrm{b}})}{{k}_{\mathrm{B}}T}\right]}{\pi {\widehat{n}}^{\mathrm{ss}}{\widehat{\eta }}_{\mathrm{max}}+{\widehat{N}}_{\mathrm{u}} \mathrm{exp}\left[\frac{{\widehat{n}}^{\mathrm{ss}}({\mu }_{\mathrm{u}}-{\mu }_{\mathrm{b}})}{{k}_{\mathrm{B}}T}\right]},$$

and ${\widehat{N}}_{\mathrm{u}}$ follows from the conservation of stress-fibre proteins throughout the cytoplasm, viz.

20 $${\widehat{N}}_{\mathrm{u}}+\frac{1}{{V}_{\mathrm{C}}}{\int }_{{V}_{\mathrm{C}}}\underset{-\pi /2}{\overset{\pi /2}{\int }}\widehat{\eta } {\widehat{n}}^{\mathrm{ss}}d\phi dV=1.$$

Thus, knowing ${\widehat{N}}_{\mathrm{u}}$ and $\widehat{\eta }\left({x}_{i},\phi \right)$, the stress ${\Sigma }_{ij}$ can now be evaluated and these stresses within the system (i.e. cell and substrate) need to satisfy mechanical equilibrium, i.e. ${\Sigma }_{ij,j}=0$. In this 2D case, the mechanical equilibrium condition is readily satisfied as the stress field ${\Sigma }_{ij}$ within the cell is equilibrated by a traction field ${T}_{i}$ exerted by the substrate on the cell such that $b{\Sigma }_{ij,j}=-{T}_{i}$, where $b({x}_{i})$ is the thickness of the cell in the current configuration. Then, ${F}_{\mathrm{cell}}$ becomes

21 $${F}_{\mathrm{cell}}\equiv {\rho }_{0}{V}_{\mathrm{C}}{\chi }_{\mathrm{u}}+{\int }_{{V}_{\mathrm{C}}}{\Psi }_{\mathrm{C}}dV +{\int }_{{V}_\mathrm{N}}{\Psi }_\mathrm{N}dV.$$

Here, ${\chi }_{\mathrm{u}}$ is given by Eq. (13) with the equilibrium value of ${\widehat{N}}_{\mathrm{u}}$ obtained from Eqs. (19–20).

### Morphological and other observables

The cell is modelled as a two-dimensional body whose morphology and position evolves on the substrate (Fig. 2). Typical observables such as 2D projections of the cell morphology and protein distributions can readily be extracted from the simulations.

#### Morphological observables

**Fig. 3:**
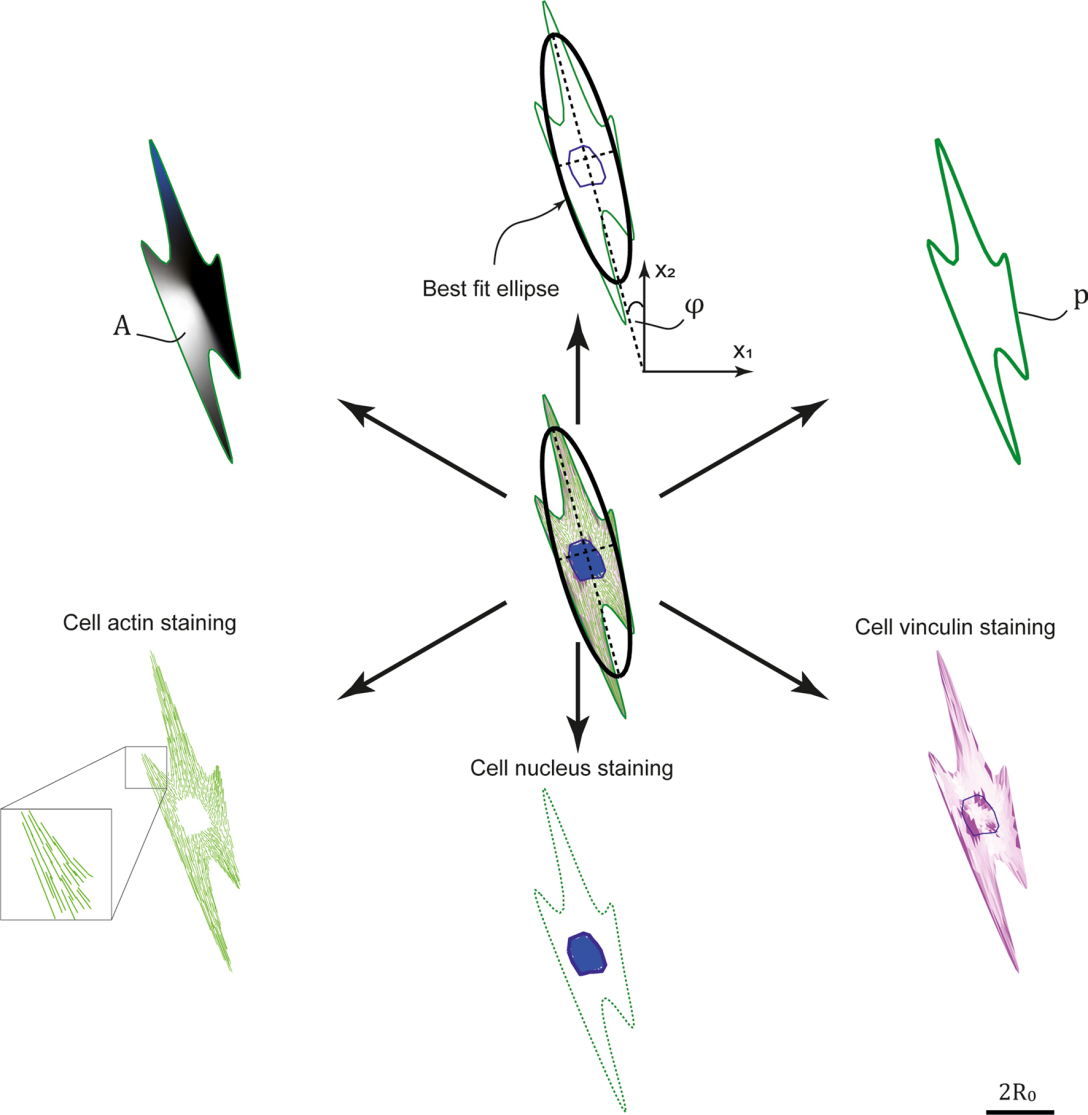
Illustrations of the cell morphological observables and staining distributions from a given morphological microstate. Scalebar is $2{R}_{0}$

The morphological observables describe the cell shape and orientation in the unpatterned or patterned substrate. These are equivalent to analysing the 2D projection of a single adherent cell on the substrate. The observables of interest, as shown in Fig. 3, are: cell area $A$, cell aspect ratio ${A}_\mathrm{S}$, orientation $\varphi$ and cell form factor $FF$. Note that all these observables are measured as a function of time.

Cell spread area $A$ is calculated as the enclosed area within the outer boundary of the cell. The cell aspect ratio ${A}_\mathrm{S}$ and orientation are defined from drawing the best fit ellipse of a given cell configuration. Note that cells take configurations that are not ellipses, but a best fit ellipse provides a metric to approximately define cell elongation and orientation and is extensively used in experiments (Buskermolen et al. 2019; Kesavan e al. 2014; Nisenholz et al. 2014; Ghibaudo et al. 2009). The best fit ellipse is calculated as an ellipse that best fits the outline of the current cell configuration. The cell aspect ratio is given by the ratio of the major to minor axis of the best-fit ellipse, and the cell orientation is the angle the major axis makes with the stripe direction. The cell form factor $\mathrm{FF}\equiv {p}^{2}/(4\pi A)$, where $p$ is the cell perimeter, is a metric typically used to describe the elongation and capability of the cell to form filopodia on a given substrate. $\mathrm{FF}$ depends on the area but gives completely different information: $\mathrm{FF}=1$ means that the cell is circular in shape while $\mathrm{FF}\gg 1$ for a starlike cell, forming many filopodia to sense and explore the environment. The perimeter $p$ is calculated as the perimeter of the polygonal shape drawn by the nodes on the periphery of the current cell configuration.

#### Visualisation of protein distributions and determining corresponding metrics

We show immunofluorescence-like images comprising of the nucleus, actin and vinculin stained in blue, green, and magenta, respectively (Fig. 3). This scheme is used for cell configurations shown throughout the paper as well as in the Supplementary Videos 1, 2 and 3. The nucleus is simply determined from colouring the mesh elements labelled as “nucleus” in blue. We proceed to discuss the protocol used to generate the protein staining for actin and vinculin.

Actin stainings are representations of the stress-fibre distributions explicitly modelled in the framework. These distributions are plotted to convey two crucial pieces of information: (i) the concentration of bound stress-fibre units in each location within the cell configuration and (ii) the local orientation of the densest stress-fibre bundle. While the formulation is a continuum formulation this information about stress-fibres is extracted from internal state variables and presented as follows. The concentration of stress-fibres $\widehat{\eta }\,\, {\widehat{n}}^{\mathrm{ss}}$ at each location ${x}_{i}$ and at an angle $\phi$ with respect to the ${x}_{2}-$ axis is known for any cell configuration. We then sketch an actin fibre in the direction ${\phi }_{\mathrm{max}}$ of the maximum value of $\widehat{\eta }\,\, {\widehat{n}}^{\mathrm{ss}}$ at that location and at a spacing that scales inversely with

22 $${\widehat{N}}_{b}\left({x}_{i}\right)=\underset{-\frac{\pi }{2}}{\overset{\frac{\pi }{2}}{\int }}\widehat{\eta } \,\,{\widehat{n}}^{\mathrm{ss}}d\phi ,$$

to convey that the actin network is more dense in that location. Focal adhesions are not explicitly modelled in the simulations. However, it is well known that mature and larger focal adhesions apply larger tractions on the substrate (Werner et al. 2017; Pelham et al. 1997; Frey et al. 2006). Thus, the magnitudes of the traction distribution $T\left({x}_{i}\right)$ are used as surrogates to visualise the localisation of the focal adhesions. Specifically, higher traction magnitudes are represented by a deeper magenta colour to visualise suggest adhesion protein density in the given location.

### Spreading of cells on substrates

It is instructive to briefly review the predictions of the homeostatic mechanics framework prior to extending the framework to predicting temporal evolutions. Recall that the homeostatic framework predicts the stationary distribution of microstates that the cell attains in a given environment. A cell in suspension needs to self-equilibrate and therefore assumes a unique configuration. For the 2D cell modelled here with the choice of parameters representing fibroblasts (Table 1), the cell in suspension is a circle of radius ${0.92R}_{0}$ with the nucleus remaining undeformed with a radius ${R}_{\mathrm{N}}$. In this configuration, the elastic stresses generated by the compression of the cytoplasm are balanced by the tensile stresses generated by a spatially uniform distribution of stress-fibres within the cytoplasm.

**Table 1:** Model parameters for myofibroblasts. In most cases, we have provided references from which these parameters were inferred. Readers are referred to Buskermolen et al. (2019) where further details are provided for all parameters

Parameter	Value [units]	Description
$T$	310 [K]	Cell temperature (37 °C)
${\mu }_{\mathrm{b}0}-{\mu }_{\mathrm{u}}$	${k}_{\mathrm{B}}T$	Difference between reference bound and unbound chemical potential Vigliotti et al. (2016)
${\sigma }_{\mathrm{max}}$	240 [$\mathrm{kPa}$]	Maximum stress-fibre tension Lucas et al. (1987)
${\stackrel{\sim }{\varepsilon }}_{\mathrm{nom}}^{\mathrm{ss}}$	0.354	Steady-state nominal strain of SF functional unit Vigliotti et al. (2016)
${\eta }_{\mathrm{max}}$	0.75	Maximum allowed angular stress-fibre density Buskermolen et al. (2019)
${\rho }_{0}$	$3\times {10}^{6}$[packets μm^−3^]	Density of protein packets that comprise stress-fibres (Vigliotti et al. 2016)
${\mu }_{\mathrm{C}}$	1.67 [$\mathrm{kPa}$]	Cytoplasm shear modulus Ronan et al. ((2012), Dowling et al. (2012 and (2013)
${m}_{\mathrm{C}}$	5	Cytoplasm shear exponent Ronan et al. ((2012), Dowling et al. (2012 and (2013)
${\kappa }_{\mathrm{C}}$	35 [$\mathrm{kPa}$]	Cytoplasm bulk modulus Ronan et al. ((2012), Dowling et al. (2012 and (2013)
${\mu }_{\mathrm{N}}$	3.3 [$\mathrm{kPa}$]	Nucleus shear modulus Ronan et al. ((2012), Dowling et al. (2012 and (2013)
${m}_{\mathrm{N}}$	20	Nucleus shear exponent Ronan et al. ((2012), Dowling et al. (2012 and (2013)
${\kappa }_{\mathrm{N}}$	35 [$\mathrm{kPa}$]	Nucleus bulk modulus Ronan et al. ((2012), Dowling et al. (2012 and (2013)
${b}_{0}/{R}_{0}$	0.05	Ratio of the cell thickness ${b}_{0}$ and cell radius ${R}_{0}$ in the elastic resting state of the cell Buskermolen et al. (2019)
${R}_{N}/{R}_{0}$	0.256 $\sqrt{\pi }$	Ratio of the nucleus radius, ${R}_\mathrm{N}$ and ${R}_{0}$ in the elastic resting state Buskermolen et al. (2019)
${\mathcal{H}}_{0}$	0.032	Volume fraction of stress-fibre proteins Vigliotti et al. (2016)
$\Omega$	${10}^{-7.1}$[$\upmu {\mathrm{m}}^{-3}$]	Volume of the reference number of functional stress-fibre units Vigliotti et al. (2016)
$\overline{\kappa }$	${10}^{5}$[$\mathrm{kPa}$]	Numerical penalty modulus
${J}_{\mathrm{c}}$	0.6	Critical value at which the elastic penalty starts to operate Shishvan et al. (2018)

**Fig. 4:**
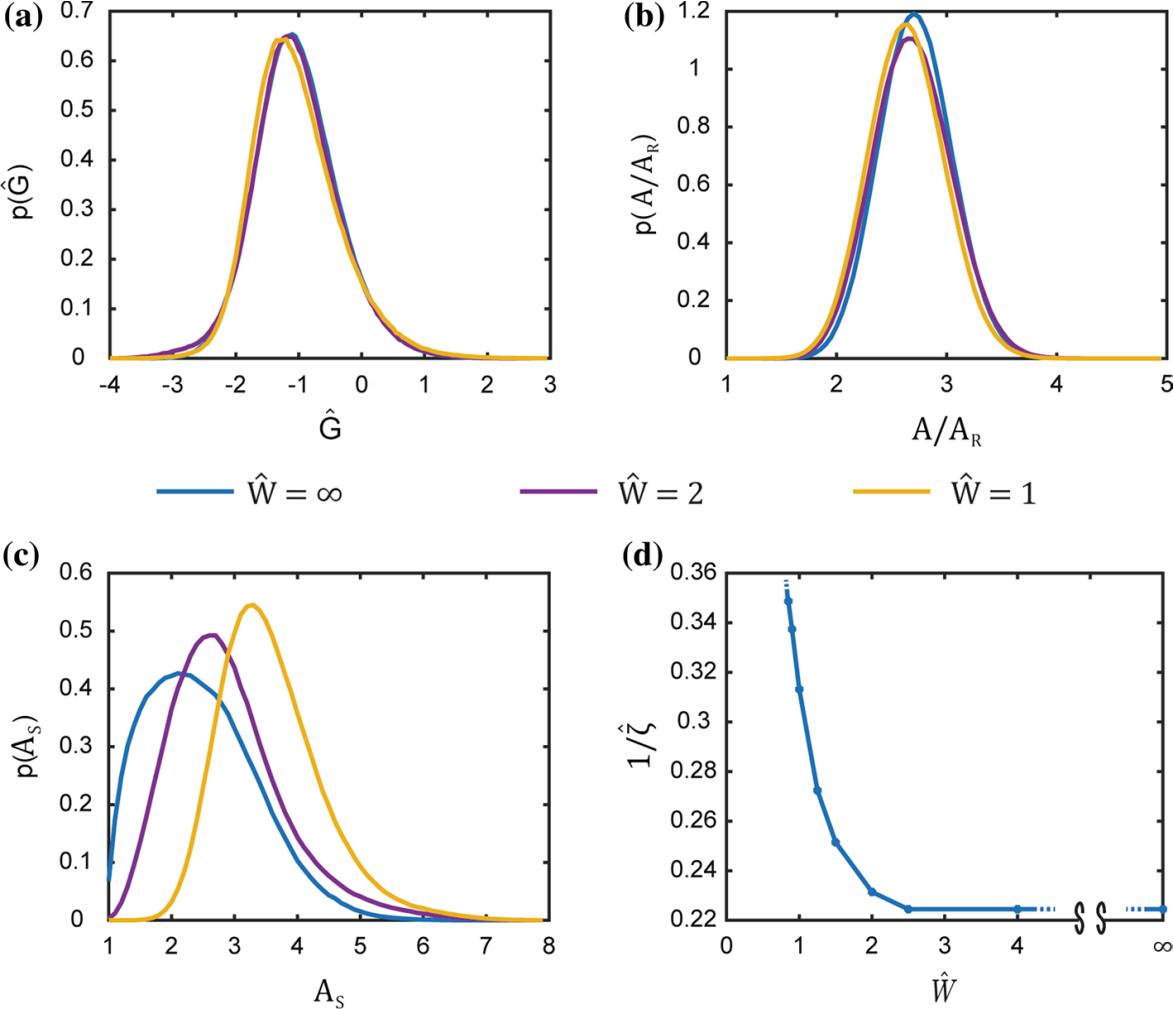
The long-term stationary response of a cell on a rigid substrate patterned with fibronectin stripes of different widths. **a**, **b**, **c** Predictions of the equilibrium probability distributions of normalised free energy $\widehat{G}$; normalised a cell area $A/{A}_\mathrm{R}$ and aspect ratio ${A}_\mathrm{S}$ for stripes of normalised widths $\widehat{W}=\infty$, i.e. fully adhesive substrate and $\widehat{W}=2$ and 1. **d** Normalised homeostatic temperature $1/\widehat{\zeta }=1/({G}_{\mathrm{S}}\zeta )$ as a function of $\widehat{W}$

Now consider a cell seeded on a patterned substrate as shown in Fig. 1b. The substrate is micropatterned with fibronectin stripes of a given width $W$, and adhesion of cell is prevented outside the stripes. This theoretical set-up is equivalent to experimentally microprinting widely spaced adhesive stripes on the substrates such that the cell cannot span two adhesive stripes and is thus confined to a single stripe. The cell stresses no longer need to be self-equilibrated as intracellular stresses can be balanced via tractions between the cell and the substrate. The cell therefore no longer attains a unique configuration but rather can fluctuate between different morphological microstates with (1) specifying the probability of a specific morphological microstate and the distribution parameter $\zeta$ set by the requirement that homeostatic constraint is satisfied over the entire ensemble of states the cell can attain. Predictions, obtained via MCMC (Appendix A), of the probability distribution of the normalised free energy $\widehat{G}\equiv G/{|G}_{\mathrm{S}}|$ of the cell are included in Fig. 4a along with the corresponding distributions of the cell area and aspect ratio in Fig. 4b and 4c, respectively, for three choices of the normalised stripe width $\widehat{W}\equiv W/(2{R}_{0})$. These metrics, typically used to characterise cell morphology, are defined as the normalised cell area $A/{A}_\mathrm{R}$ where $A$ and ${A}_\mathrm{R}$ are the areas of the cell and the area of the cell in suspension (${A}_\mathrm{R}=\pi {\left(0.92{R}_{0}\right)}^{2}$), respectively. Clearly, the cells are predicted to spread and elongate on the substrate with the driving force for these morphological changes arising from the fact that these spread and elongated states have low free energy: as discussed by Shishvan et al. (2018), spreading of the cell reduces its cytoskeletal free energy but results in an increase in the elastic energy and it is this competition that controls cell spreading. We observe that the confinement imposed by the adhesive stripes has a strong tendency to elongate the cells but has a minimal effect on the distribution of the cell free energy and cell area. It is important to recognise here that cells do not uniquely attain their lowest free-energy state but rather sample a wide distribution of morphological states as evidenced in Fig. 4b and 4c. This wide sampling is set by the distribution parameter $\zeta$ as required to satisfy the homeostatic constraint. In fact, the model predicts that the normalised homeostatic “temperature” $1/\widehat{\zeta }=1/({G}_{\mathrm{S}}\zeta )$ increases with decreasing $\widehat{W}$ as seen in Fig. 4d. These results are consistent with the predictions in Buskermolen et al. (2019) and serve as a useful reference as we proceed to understand the temporal evolution of cells seeded on substrates.

## A Langevin style framework for cell dynamics

The homeostatic mechanics framework gives the stationary distribution (1) of morphological microstates that cells will attain in a given environment as seen above. This stationary state is typically attained within 24 to 36 h after seeding cells into that environment. The framework gives no temporal information about the evolution of cells as they attain this stationary state including whether all morphological observables reach their stationary distribution at the same rate or whether there are multiple operative timescales. Here, we propose the simplest possible extension of the homeostatic mechanics framework to a dynamical setting by invoking an analogy with the canonical ensemble.

Within the context of the homeostatic ensemble, the Gibbs free energy ${G}^{\left(j\right)}$ of a cell fluctuates while the cell is in its stationary state (also referred to as homeostatic equilibrium), but the corresponding homeostatic potential $\mathcal{M}\equiv \overline{G } -(1/\zeta ){S}_{\Gamma }$ (Shishvan et al. 2018) remains constant, where $\overline{G}\equiv \langle {G }^{\left(j\right)}\rangle$ and ${S}_{\Gamma }$ the morphological entropy of the cell. Thus, a direct analogy can be made between the homeostatic ensemble and the well-established canonical ensemble where the Helmholtz free energy $F\equiv \overline{U }-TS$ is a constant in a bath with a thermodynamic temperature $T$. In this bath, the system fluctuates over its microstates $(j)$ such that it has a fluctuating internal energy ${U}^{\left(j\right)}$ which at equilibrium achieves average value $\overline{U}\equiv \langle {U }^{\left(j\right)}\rangle$ and entropy $S$. Thus, we see that the internal energy ${U}^{\left(j\right)}$ and temperature $T$ in the canonical ensemble are analogous to ${G}^{\left(j\right)}$ and $1/\zeta$, respectively, in the homeostatic ensemble. The temporal evolution of the microstates of an isothermal system whose equilibrium is given by the canonical ensemble is often described by Langevin dynamics. The low speeds at which cells move (Purcell 1977) imply that it suffices to ignore inertia and overdamped Langevin dynamics for the homeostatic ensemble, which we call Homeostatic Langevin Equation (HLE), can be developed as follows. We specify that the temporal evolution of the co-ordinates $\tilde{r }$ that describe the cell morphology is given by

23 $$\frac{{\partial {}_{{}}^{\left( q \right)} r_{i} }}{\partial t} = \frac{1}{\gamma }{}_{{}}^{\left( q \right)} {\mathcal{F}}_{i} \left( {\tilde{r},t} \right) + f_{i} \left( t \right),$$

where $t$ is time and $\gamma$ is a damping coefficient, sometimes referred to as the mobility, that relates the velocity of the microvariable ${}_{{}}^{\left( q \right)} r_{i}$ to a determinstic force

24 $${}_{{}}^{\left( q \right)} {\mathcal{F}}_{i} \left( {\tilde{r},t} \right) \equiv - \frac{{\partial G\left( {\tilde{r}} \right)}}{{\partial {}_{{}}^{\left( q \right)} r_{i} }}$$

and ${f}_{i}\left(t\right)$ a contribution from a random force. Thus, while the first term on the right-hand-side of (23) represents a deterministic force contribution, the second term is the random contribution associated with large non-thermal fluctuations due to high-energy nutrient exchanges between the cell and the nutrient bath in which it resides. In principle, ${f}_{i}(t)$ are correlated in time since the molecular processes from which they originate have a finite correlation times (Roberts et al. 2014). However, observations (Stokes et al 1991; Roberts et al. 2014) suggest diffusive type behaviour of cells over timescales of a few minutes and thus we assume that there exists some correlation timescale ${t}_{c}$ over which $\langle f_{i} \left( t \right)f_{j} \left( {t}^\prime \right)\rangle = g_{ij} \left( {t - t^\prime } \right)$ decays rapidly. Then, on timescales $\gg {t}_{c}$ (which in the case of fibroblasts is on the order of a few minutes), ${f}_{i}\left(t\right)$ can be taken to be a delta correlated stationary Gaussian process satisfying $\langle {f}_{i}(t)\rangle =0$ and $\langle f_{i} \left( t \right)f_{j} \left( {t^\prime } \right)\rangle = \xi \delta_{ij} \delta \left( {t - t^\prime } \right)$ where ${\delta }_{ij}$ and $\delta (\bullet$) are Kronecker and Dirac deltas, respectively, and $\upxi$ the standard deviation of the random and decorrelated ${f}_{i}\left(t\right).$ Under these assumptions, (23) reduces to the Langevin equation. Thus, the HLE is valid for observation times scales $\gg {t}_{c}$ (i.e. a vanishing Deborah number) and only captures the diffusive nature of cell motility but not the ballistic response of cells which occurs on the scale of a few minutes.

It now remains to set the standard deviation $\upxi$. In order to set $\upxi$, we turn to the Fokker–Planck equation corresponding to the HLE. In the HLE, we expressed the uncertainty in the morphology of the cell in terms of Gaussian correlation functions. Shifting perspective, we can ask: what probability distribution $P\left(\tilde{r },t, {\tilde{r }}_{0}\right)$, where ${\tilde{r }}_{0}$ is $\tilde{r }$ at time $t=0$, would give the same correlation functions? It is important here to stress that we do not care about that path $\tilde{r }(t)$ the cell took but rather ask the simpler question of the probability that the cell attains a morphological microstate $\tilde{r }(t)$ at time $t$, regardless of how it got there. This is equivalent to saying that if we run a large number of independent but nominally identical experiments then what is the probability distribution of morphological microstates observed at time $t$. This is a well-established problem and following Ichimaru (2018), the required probability distribution can be shown to satisfy the Fokker–Planck equation

25 $$\frac{{\partial P\left( {\tilde{r},t} \right)}}{\partial t} = \frac{1}{\gamma }\mathop \sum \limits_{i = 1}^{2} \mathop \sum \limits_{q = 1}^{M} \frac{\partial }{{\partial {}_{{}}^{\left( q \right)} r_{i} }}\left( {P\left( {\tilde{r},t} \right)\frac{{\partial G\left( {\tilde{r}} \right)}}{{\partial r_{i} }}} \right) + \frac{{{\upxi }^{2} }}{2}\mathop \sum \limits_{i = 1}^{2} \mathop \sum \limits_{q = 1}^{M} \frac{{\partial^{2} P\left( {\tilde{r},t} \right)}}{{\partial {}_{{}}^{\left( q \right)} r_{i}^{2} }} .$$

The steady-state solution to (25) corresponding to $\partial P\left(\tilde{r },t\right)/\partial t=0$ is the equilibrium probability distribution and given by:

26a $${P}_{\mathrm{eq}}\left(\tilde{r }\right)=\frac{1}{Z}\mathrm{exp}\left[-\frac{2}{\gamma {\upxi }^{2}}G\left(\tilde{r }\right)\right],$$

where

26b $$Z\equiv \int \mathrm{exp}\left[-\frac{2}{\gamma {\upxi }^{2}}G\left(\tilde{r }\right)\right]d\tilde{r }.$$

Therefore, the Fokker–Planck Eq. (25) converges to the homoeostatic ensemble (1) by setting:

27 $$\upxi =\sqrt{\frac{2}{\gamma \zeta }}.$$

We shall thus use this choice of $\upxi$ to resolve the temporal response of a single cell in a given environment. Recalling that $f_{i} \left( t \right)$ in (23) is random and decorrelated with a standard deviation ${\upxi }$ we can rewrite (23) in normalised form as:

28 $$\frac{{\partial {}_{{}}^{\left( q \right)} \hat{r}_{i} }}{{\partial \hat{t}}} = - \frac{{\partial \hat{G}}}{{\partial {}_{{}}^{\left( q \right)} \hat{r}_{i} }} + \sqrt {\frac{2}{{\hat{\zeta }{\Delta }\hat{t}}}} {\mathcal{N}}\left( {0,1} \right),$$

where ${}_{{}}^{\left( q \right)} \hat{r}_{i} \equiv {}_{{}}^{\left( q \right)} r_{i} /R_{0}$, $\hat{G} \equiv G/\left| {G_{{\text{S}}} } \right|$, $\hat{\zeta } \equiv \zeta \left| {G_{{\text{S}}} } \right|$ and $\hat{t} \equiv t\left| {G_{{\text{S}}} } \right|/\left( {\gamma R_{0}^{2} } \right)$ while ${\mathcal{N}}\left( {0,1} \right)$ is a Gaussian distribution of zero mean and unit variance. In writing (28), we have used (27) and the fact that the stochastic differential Eq. (28) is solved with a finite time step $\Delta t$ with$\Delta \widehat{t}\equiv\Delta t|{G}_{\mathrm{S}}|/(\gamma {R}_{0}^{2})$. It is now apparent that while $\zeta$ sets the fluctuation magnitude, the mobility $\gamma$ sets the evolution timescale. We emphasise that this single timescale for the evolution of the morphological microstate does not imply that all observables evolve at the same rate as we shall proceed to show. All results are presented for model parameters representative of myofibroblasts as calibrated in Buskermolen et al. (2019), but we emphasise that the model holds more generally for any adherent single cell type.

### Temporal integration of the HLE

The stationary distributions for the observables as well as the homeostatic temperature used in the dimensionless form of the HLE (28) were provided by MCMC calculations; see Appendix A. Stochastic differential equations are typically integrated using a Euler forward integration algorithm (Milstein 1994) which here was employed as follows:

(i) The cell is placed from suspension on the origin of the substrate at time $\widehat{t}=0$. On the adhesive stripes the origin coincides with the axis of the stripe.
(ii) For the microstate at time $\widehat{t}$ described by the vectors ${}^{\left(q\right)}{\widehat{r}}_{i}(\widehat{t})$, we evaluate the gradient $\partial \widehat{G}/ \partial {}^{\left(q\right)}{\widehat{r}}_{i}$ using the function “Derivest” (D’Errico 2007). Derivest is a complex numerical differentiation algorithm that requires 46 separate evaluations of $\widehat{G}\left(\tilde{r }\right)$; readers are referred to D’Errico (2007) for details of the algorithm.
(iii) For all the degrees of freedom we then randomly select the value of the noise from the normal Gaussian distribution, $\mathcal{N}(\mathrm{0,1})$ and insert it into (28) to obtain the value of $\partial {}^{\left(q\right)}{\widehat{r}}_{i}/\partial \widehat{t}$.
(iv) We then update the current configuration by via the forward Euler scheme.
  29 $${}_{{}}^{\left( q \right)} \hat{r}_{i} \left( {\hat{t} + \Delta \hat{t}} \right) = {}_{{}}^{\left( q \right)} \hat{r}_{i} \left( {\hat{t}} \right) + \frac{{\partial {}_{{}}^{\left( q \right)} \hat{r}_{i} }}{{\partial \hat{t}}}\Delta \hat{t}.$$
(v) All the observables associated to the new cell configuration ${}^{\left(q\right)}{\widehat{r}}_{i}\left(\widehat{t}+\Delta \widehat{t}\right)$ are then stored.
(vi) Set $\widehat{t}=\widehat{t}+\Delta \widehat{t}$, and repeat from step (ii) until $\widehat{t}={\widehat{T}}_{\mathrm{Sim}}$, where ${\widehat{T}}_{\mathrm{Sim}}$ is the maximum simulated time of the experiment.

Time evolutions in both the unpatterned and patterned substrates were computed by selecting $\Delta \widehat{t}=0.001$. Numerical convergence checks confirmed that reducing the time step further did not result in any changes to the predictions. Moreover, at steady state all observables predicted by the HLE using this value of the time-step coincided with the MCMC calculations. All algorithms, except for Derivest, were developed in-house in Matlab and the typical computational duration for a single HLE trajectory of duration ${\widehat{T}}_{\mathrm{Sim}}=4000$ using 8 parallel CPUs is around 20 days.

## Cell area and elongation evolve at different rates on unpatterned substrates

We shall discuss two cases: (i) cells on unpatterned substrates where there is effectively no confining effect imposed by the substrate within the ${x}_{1}-{x}_{2}$ plane and then proceed to contrast with the case of (ii) contact guidance on substrates patterned with adhesive stripes of width $W$ (Fig. 1b).

**Fig. 5:**
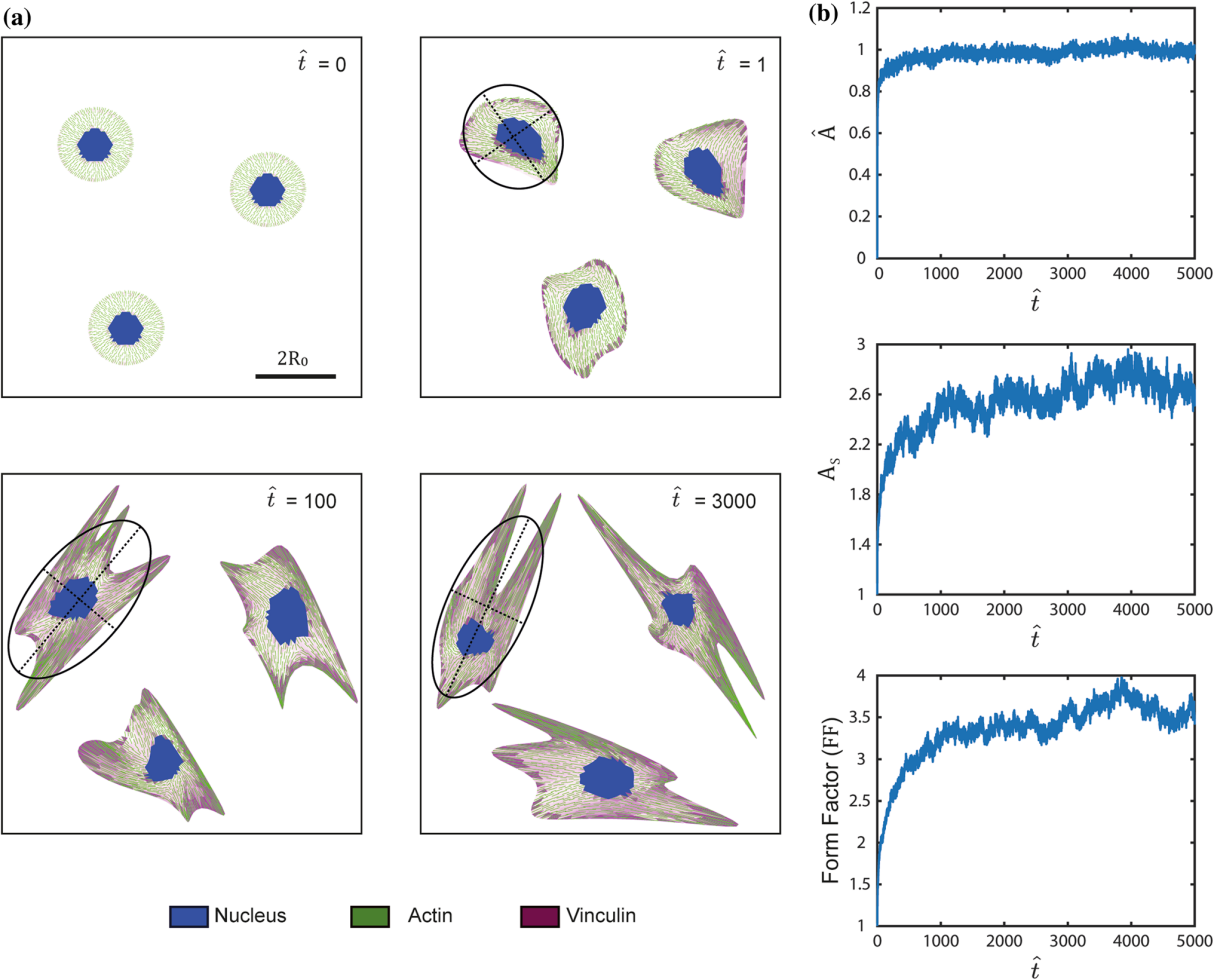
Temporal evolution of cells on unpatterned substrates. **a** Three representative images of cells at three normalised times $\widehat{t}$ for cells seeded at time $\widehat{t}=0$. An example of a best fit ellipse is drawn on one of the selected temporal evolution. The scalebar is $2{R}_{0}$. **b** Temporal evolution of morphological observables, viz. normalised cell area $\widehat{A}$, aspect ratio ${A}_\mathrm{S}$ and form factor $\mathrm{FF}$. The results are shown as averages over $n=100$ Langevin trajectories

We conceive of a typical experiment where a cell in suspension is seeded on a rigid substrate coated uniformly with an adhesive protein such as fibronectin. The evolution of the cell morphology is then observed as a function of time $t$, with $t=0$ corresponding to the instant of seeding. Of course, motility of the cell and the evolution of its morphology are coupled, but first we focus on cell morphology. Representative images of computed cell morphologies and the corresponding actin, nucleus and focal adhesion organisations are included in Fig. 5a at three normalised times $\widehat{t} = 1, 100, 3000$ in addition to the state at $\widehat{t}=0$. Recall that the Langevin style dynamical Eq. (28) is a stochastic differential equation so that a different solution is generated for every realisation of the noise process, i.e. much like in experiments a different trajectory of morphological evolution is obtained for every solution of (28) with the same initial state at $t=0$. Hence, in Fig. 5a we show solutions at three times from three such trajectories. While the three morphologies computed using different trajectories of (28) are different, they show many similar features. These include the observations that with increasing time: (i) the cells spread and increase their area as well as their ellipticity or aspect ratio and (ii) the level of actin polymerisation and focal adhesions also increase.

It is instructive to make quantitative predictions of some morphological observables to enable more direct comparison with measurements. The complete morphology of the cell in the model is described by ${\tilde{r }}^{(j)}$, but as in observations (Buskermolen et al. 2019) we focus on three coarse-grained metrics of the morphology, viz. (i) cell area $A$, (ii) cell aspect ratio ${A}_\mathrm{S}$ and (iii) the form factor $\mathrm{FF}$. Each solution of the HLE (28) produces a different trajectory, and much like in experiments, trends are most clearly seen by examining the ensemble average over a number of trajectories. We performed $n=100$ independent simulations and defined the ensemble average as follows. At time $t$, the ensemble average area is defined as $\overline{A }(t)=(1/n){\sum }_{i=1}^{n}{A}_{(i)}(t)$, where ${A}_{(i)}(t)$ is the area of the cell in the ${i}^{\mathrm{th}}$ trajectory at time $t$. The ensemble average aspect ratio ${\overline{A} }_\mathrm{S}(t)$ and $\overline{\mathrm{FF} }(t)$ are defined in an analogous manner. While ${A}_\mathrm{S}$ and $\mathrm{FF}$ are non-dimensional, it is instructive to define a non-dimensional area as

30 $$\hat{A}(t) \equiv \frac{{\overline{A}(t) - A_\mathrm{R} }}{{A_{\infty } - A_\mathrm{R} }}$$

where ${A}_{\infty }\equiv \overline{A }(t\to \infty )$ so that $\widehat{A}$ assumes values of 0 when the cell is seeded and fluctuates around 1 at convergence. Predictions of the temporal evolution with normalised time $\widehat{t}$ are included in Fig. 5b and are consistent with the qualitative features seen in images of the cell morphologies (Fig. 5a). However, an intriguing feature emerges: while the HLE has only a single timescale, the normalised cell area $\widehat{A}$ evolves and reaches its steady state much faster compared to the cell aspect ratio and form factor, i.e. different timescales for the evolution of area and shape emerge from the HLE. See Supplementary Video 1 for the simulated evolution of the cell over a single trajectory shown alongside the plots of the evolution of the ensembled averaged observables.

**Fig. 6:**
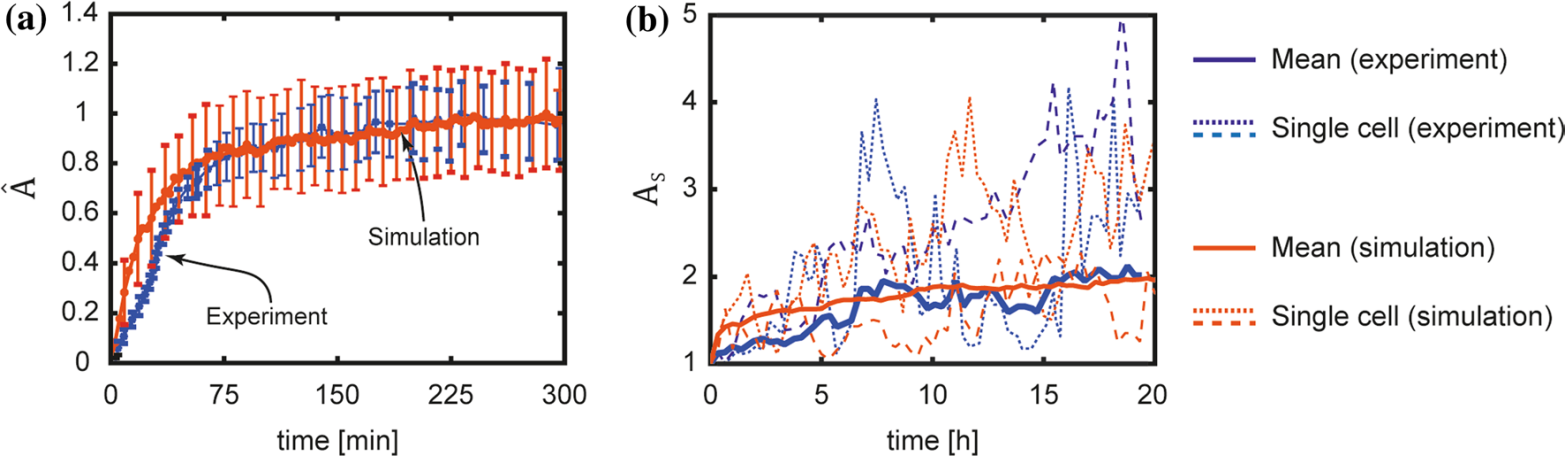
Timescales for the evolution of cell morphology. **a** Comparison between measurements (Nisenholz et al. 2014) and predictions of the temporal evolution of normalised cell area $\widehat{A}$. Solid lines and error bars indicate the average and standard deviation, respectively, over $n=50$ experimental measurements and $n=100$ Langevin trajectories of the simulations. **b** Corresponding comparisons between measurements (Kesavan et al. 2014) and simulations of the cell aspect ratio ${A}_\mathrm{S}$. The solid lines are the average over $n=10$ experimental measurements and $n=100$ Langevin trajectories of the simulations, while the dotted lines show selected trajectories in the measurements and simulations to indicate the wide variability in both the measurements and simulations. The simulations use a timescale $(\gamma {R}_{0}^{2})/|{G}_{\mathrm{S}}|=10 \mathrm{min}$. This single timescale is shown to capture the observation that the cell area evolves in minutes, while the cell aspect ratio evolves over a timescale of many hours

The different timescales for the evolution of cell area and aspect ratio have also been observed in experiments. In Fig. 6a and 6b, we reproduce measurements from Nisenholz et al. (2014) and Kesavan et al. (2014) of the temporal evolution of normalised area and aspect ratio, respectively, for the spreading of fibroblasts on stiff substrates with $t=0$ corresponding to the instant of seeding the cell from suspension. The experimental data for area were normalised following (30) while the aspect ratio was inferred from the circularity data reported by Kesavan et al. (2014). In Fig. 6a, the average cell area over about 50 measurements is plotted with the error bars corresponding to the standard deviation, while in Fig. 6b the average aspect ratio is plotted over 10 measurements (two measured trajectories are also included to give an indication of the large variability in this metric over different trajectories). The striking difference between Fig. 6a and 6b is that while the steady-state cell area is achieved in about 150 min the cell aspect ratio evolves significantly more slowly such that is unclear whether a steady-state aspect ratio is achieved 20 h after seeding. We superimpose on Fig. 6 our corresponding predictions (averaged over 100 different trajectories) with the timescale of in the simulations chosen to be $(\gamma {R}_{0}^{2})/|{G}_{\mathrm{S}}|=10 \mathrm{min}$ so as to match the simulated and measured timescales of the temporal evolution of cell area. With this choice of the timescale we see that the simulations capture the observation that the cell aspect ratio evolves significantly more slowly. Moreover, the simulations not only capture the average temporal evolution response but also agree with measurements of the standard deviation of cell area. In addition, two selected simulated aspect ratio trajectories are included in Fig. 6b and illustrate that, in line with measurements, the simulations also display a very large variability of this metric over different trajectories.

**Fig. 7:**
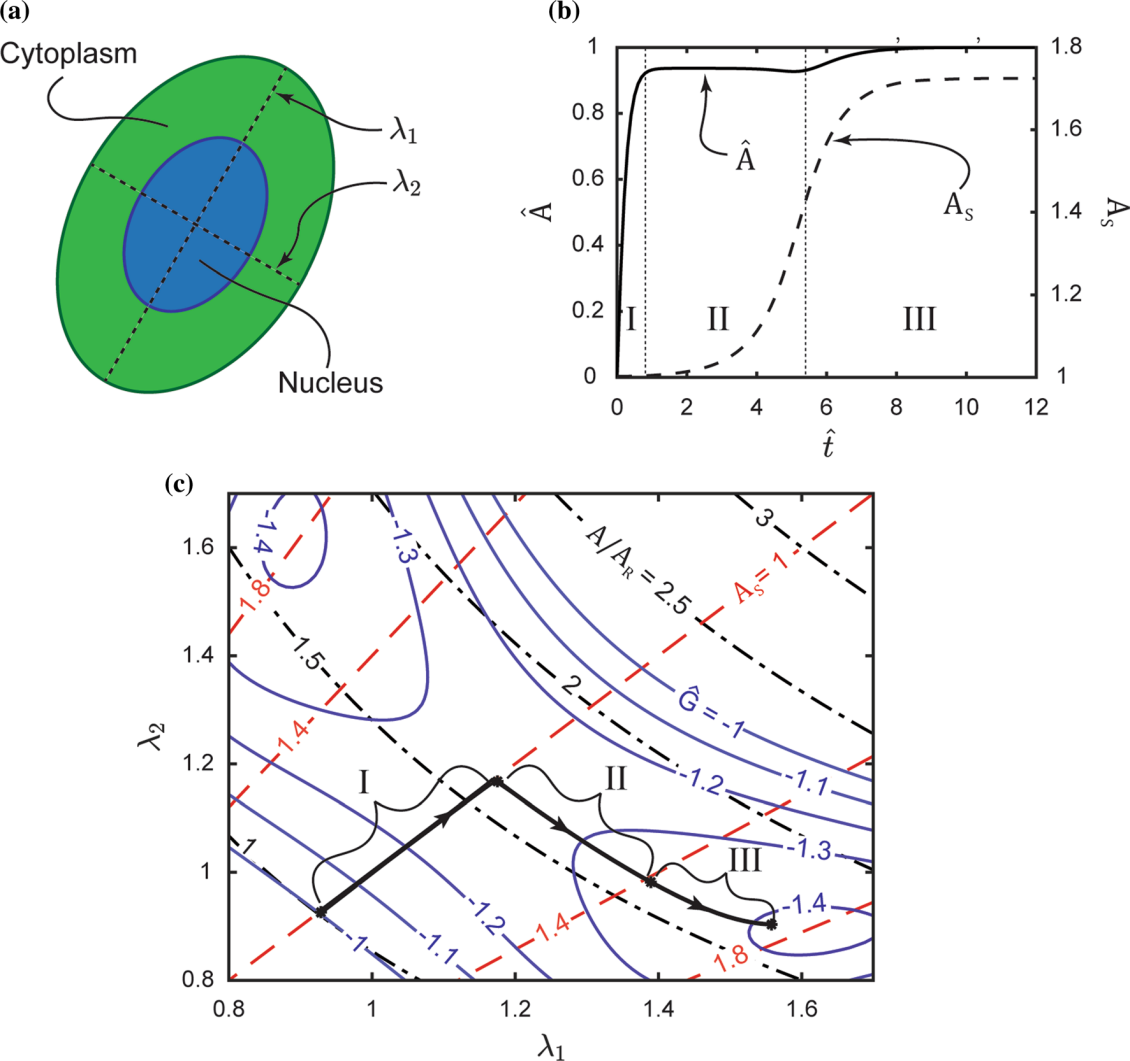
A reduced model of a cell as a spatially uniform ellipse to illustrate morphological evolution. **a** Sketch of the cell including its nucleus as a spatially uniform ellipse. Here, ${\lambda }_{1}$ and ${\lambda }_{2}$ are the stretches of the axes of the ellipse. **b** Predictions of the temporal evolution of the normalised ellipse area and aspect ratio ${A}_\mathrm{S}\equiv {\lambda }_{1}/{\lambda }_{2}$ (${\lambda }_{1}\ge {\lambda }_{2}$) for a cell seeded on an unpatterned substrate from suspension at time $\widehat{t}=0$. The predictions are for a deterministic response with effects of biological noise excluded. **c** Free-energy landscape of the reduced cell model. Contours of the normalised free energy $\widehat{G}$ are included on map with axes ${\lambda }_{1}$ and ${\lambda }_{2}$ along with contours of $A/{A}_\mathrm{R}$ and ${A}_\mathrm{S}$. On the map, we show the deterministic trajectory of the cell in the free-energy landscape for a cell starting from its state in suspension until it attains its minimum free-energy state on the substrate

The success of the simulations in capturing the fact that the two morphological observables evolve at different rates suggests that a single rate constant $(\gamma {R}_{0}^{2})/|{G}_{\mathrm{S}}|$ suffices to set the evolution of these observables. We therefore use the model to interrogate the source of the two timescales that set the evolution of cell area and aspect ratio. For this, we consider a significantly simplified model where the cell is restricted to remain a spatially uniform ellipse with a fixed orientation (Fig. 7a). In this case, the stretches ${\lambda }_{1}$ and ${\lambda }_{2}$ of the principal axes of the ellipse completely define the cell morphology rather than the $M$ positional vectors ${}^{\left(q\right)}{\widehat{r}}_{i}$. The deterministic evolution of the cell morphology in this simplified model is then given by an equation analogous to (28) with the noise term neglected, viz.

31 $$\frac{\partial {\lambda }_{i}}{\partial \widehat{t}}=-\frac{\partial \widehat{G}}{\partial {\lambda }_{i}},$$

where $i=1, 2$. Predictions of the temporal evolution of $\widehat{A}$ and ${A}_\mathrm{S}$ (where cell area $A={\lambda }_{1}{\lambda }_{2}{\pi}{R}_{0}^2$ and ${A}_\mathrm{S}={\lambda }_{1}/{\lambda }_{2}$ with ${\lambda }_{1}\ge {\lambda }_{2}$) are included in Fig. 7b for a cell seeded from suspension at $\widehat{t}=0$. Intriguingly, the qualitative feature that the cell area attains its steady state significantly faster than aspect ratio is retained in this very simplistic setting.

We observe three temporal regimes: (i) an initial regime I of rapid cell spreading where the cell remains circular with ${A}_\mathrm{S}=1$; (ii) a subsequent regime II of cell elongation where cell area is constant but the aspect ratio increases and (iii) a final regime III where both cell area and aspect ratio increase although the changes in this regime are relatively minor. Regimes I and II set the two observed timescales for the evolution of cell area and aspect ratio, respectively. In this deterministic and simplistic setting where the cell morphology is only a function of $({\lambda }_{1},{\lambda }_{2})$, we can understand this by examining the energy landscape $\widehat{G}\left({\lambda }_{1},{\lambda }_{2}\right)$ shown in Fig. 7c. We include in Fig. 7c the trajectory the cell takes in $({\lambda }_{1},{\lambda }_{2})$ space starting from its state in suspension along with isolines of $A/{A}_\mathrm{R}={\lambda }_{1}{\lambda }_{2}/(0.92)^2$ and ${A}_\mathrm{S}={\lambda }_{1}/{\lambda }_{2}$. The trajectory set by Eq. (31) has two distinct branches: (i) an initial branch corresponding to regime I where the cell traverses a path of ${A}_\mathrm{S}=1$ while $A$ increases and then (ii) turns and traverses a path of constant $A$ but with increasing ${A}_\mathrm{S}$. This trajectory is purely set by the topology of the $\widehat{G}({\lambda }_{1},{\lambda }_{2})$ landspace and the fact that (31) requires the cell morphology to evolve along a path with the steepest gradient in $\widehat{G}({\lambda }_{1},{\lambda }_{2})$ space. Thus, we argue that the two different timescales for the evolution of cell area and aspect ratio are purely a result of the free-energy landscape of the cell. This landscape is set by the interplay between the elastic energy and cytoskeletal energy of the cell.

### Diffusive motility of cells on unpatterned substrates

**Fig. 8:**
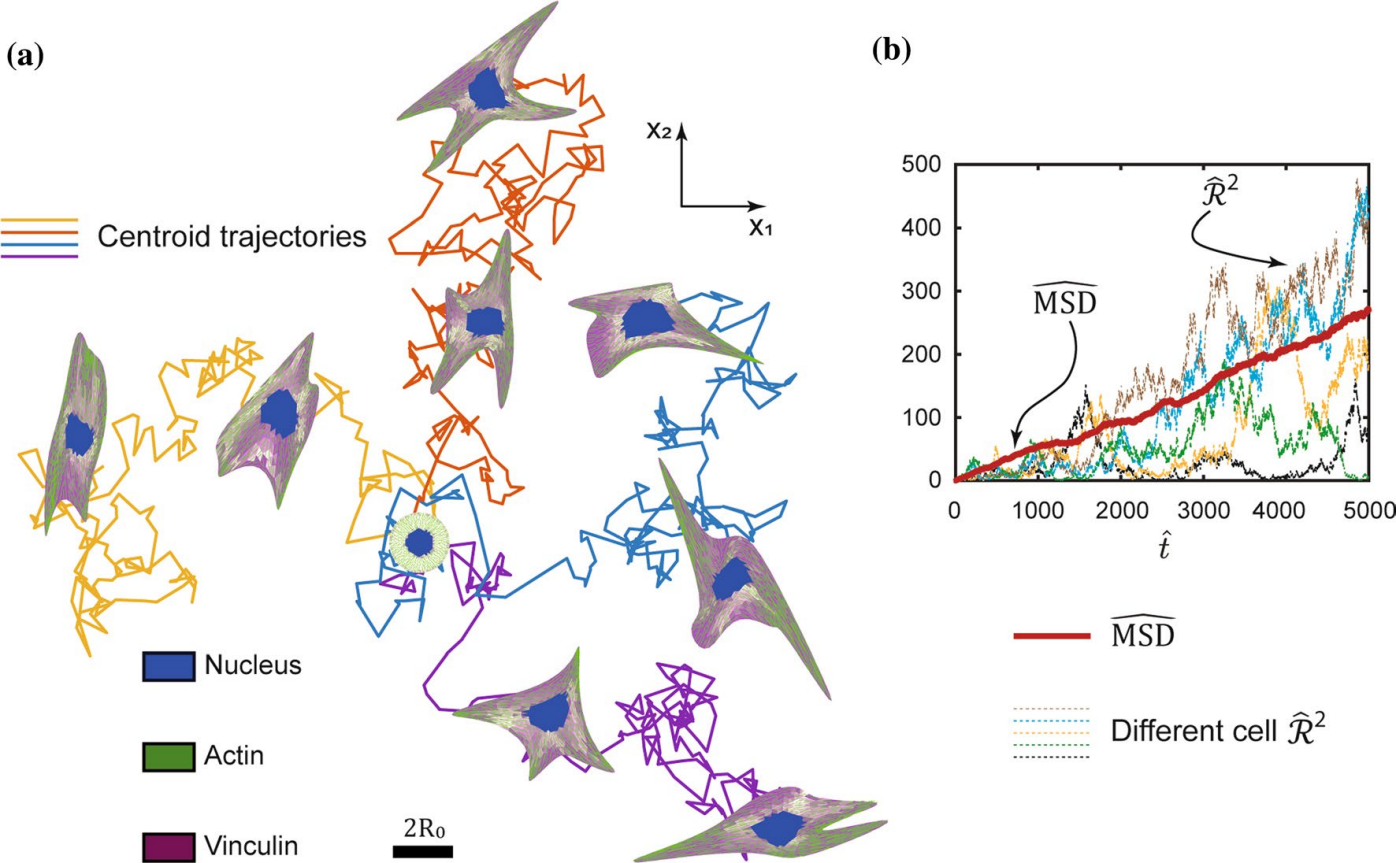
Coupled motility and morphological evolution of the cell seeded on an unpatterned substrate.** a** Four Langevin trajectories for a cell seeded at time $\widehat{t}=0$ from suspension on an unpatterned substrate. The scalebar $2{R}_{0}$ is the diameter of the circular cell in its elastic rest state.** b** Predictions of the corresponding temporal evolution of the normalised mean square displacement $\widehat{\mathrm{MSD}}$ over $n=100$ Langevin trajectories. Four indivual trajectories parameterised by their normalised squared displacement ${\widehat{\mathcal{R}}}_{\left(k\right)}^{2}$ are also included to illustrate the variability in the trajectories as seen in (a)

When cells are seeded on substrates coated with an adhesive protein, not only does their morphology evolve but this morphological evolution is coupled to their motility. The HLE (28) enables predictions of this coupled motility and morphological evolution of the cell. Predictions of the coupled evolution of the cell morphology and its motility are shown in Fig. 8 for 4 selected trajectories of (28) with the cell seeded from suspension at same location at time $\widehat{t}=0$ in each case. Stochastic motility (i.e. cells take a random path over the substrate surface) is predicted over the timescales in Fig. 8a in line with numerous observations (Stokes et al. 1991; Plou et al. 2018; Dunn and Brown 1987; Schienbein and Gruler 1993; Krummel et al. 2016). The stochastic motility can be characterised in terms of the squared displacement

32 $${\mathcal{R}}_{\left(k\right)}^{2}(t)={x}_{\left(k\right)}^{2}(t)+{y}_{\left(k\right)}^{2}(t),$$

of the centroid of cell $(k)$ from its seeding location (taken to be the origin of the Cartesian co-ordinate system with ${x}_{\left(k\right)}(t)$ and ${y}_{\left(k\right)}(t)$ the Cartersian co-ordinates of the cell centroid at time $t$, where $x\equiv {x}_{1}$ and $y\equiv {x}_{2}$). Predictions of ${{\widehat{\mathcal{R}}}_{\left(k\right)}^{2}\equiv \mathcal{R}}_{\left(k\right)}^{2}(\widehat{t})/{R}_{0}^{2}$ are included in Fig. 8b for 5 trajectories: we predict large variations in ${\widehat{\mathcal{R}}}_{\left(k\right)}^{2}$ over different trajectories consistent with observations (Stokes et al. 1991). (The time-lapse of the motility of a single trajectory of a cell along with the corresponding $\widehat{\mathcal{R}}(\widehat{t})$ is included in Supplementary Video 2.) Given this stochastic nature of the motility, it is more instructive to consider the mean squared displacement $\widehat{\mathrm{MSD}}\equiv (1/n){\sum }_{k=1}^{n}{\widehat{\mathcal{R}}}_{\left(k\right)}^{2}$ over $n$ independent trajectories. The predictions of $\widehat{\mathrm{MSD}}$ ($n=100$) are included in Fig. 8b and illustrate the diffusive nature of the predicted motility as $\widehat{\mathrm{MSD}}$ scales linearly with normalised time $\widehat{t}$. If the cell is assumed to be a single particle whose position is given by the cell centroid, the HLE (28) would predict a diffusion coefficient $D=1/(\gamma \zeta )$ such that the slope of the $\widehat{\mathrm{MSD}}$ curve in Fig. 8b is given by the normalised diffusion coefficient $\widehat{D}\equiv D\gamma /|{G}_{\mathrm{S}}|=1/(\zeta |{G}_{\mathrm{S}}|)$. (The HLE in this context describes the Brownian motion of the cell with the homeostatic temperature setting the magnitude of the fluctuations.) The $\widehat{\mathrm{MSD}}$ slope in Fig. 8b $\approx 0.06$, while for the cell parameters used here $\widehat{D}\approx 0.23$, i.e. the actual motility of the cell as predicted by (28) is significantly more sluggish compared to modelling the cell as a Brownian particle with a diffusion coefficient $\widehat{D}$. The slower motion is related to the morphological evolution of the cell that is coupled to its motility. If the cell were modelled as a single Brownian particle, all fluctuations would result in motion of the particle. On the other hand, in (28), the fluctuations affect the positional vectors ${}_{{}}^{\left( q \right)} r_{i}$ $(i=\mathrm{1,2})$ of the $q=1,\dots ,M$ that describe the cell morphology and a displacement of the cell centroid requires the co-ordinated motion of these vectors. Such co-ordinated motion via stochastic fluctuations occurs with a low probability resulting in the effective diffusion coefficient of the cell centroid being significantly smaller than $\widehat{D}$.

Finally, we emphasise that our model captures the coupled evolution of cell morphology and motility over timescales ${\gg t}_{c}$, i.e. we only capture the diffusive motion of the cell and not its ballistic motion (Dunn and Brown 1987) due to persistence in the direction of cell motion over timescales ${\ll t}_\mathrm{c}$. A consequence of the model only capturing the diffusive part of the motion is that it does not provide insights into the physical processes, such as actin treadmilling, that give rise to ballistic motion (Roberts et al. 2014; Lee et al. 2021; Recho et al. 2013; Cardamone et al. 2011).

## Contact guidance on substrates with adhesive stripes

In vivo, adherent cells interact with the surrounding extracellular matrix (ECM) that is not only responsible for the structural integrity of tissues but also establishes and maintains the cellular microenvironment by providing cells with mechanical, biochemical and physical cues. It is now well established that cellular microenvironments induce cells to align and migrate along the direction of the anisotropy—a phenomenon called contact guidance (Chang et al. 2013). Various in vitro chemical micropatterning approaches using two-dimensional (2D) substrates have been developed to study cellular contact guidance, as model systems to simplify the highly complex three-dimensional (3D) environments in vivo (Buskermolen et al. 2019). A common micropattern is fibronectin stripes of a given width $W$ with adhesion of cell prevented outside the stripes (Buskermolen et al. 2019; Buskermolen et al. 2020). We now proceed to investigate via the HLE the effect of the confinement provided by the stripes on the evolution of cell morphology and the accompanying motility which finally leads to contact guidance.

**Fig. 9:**
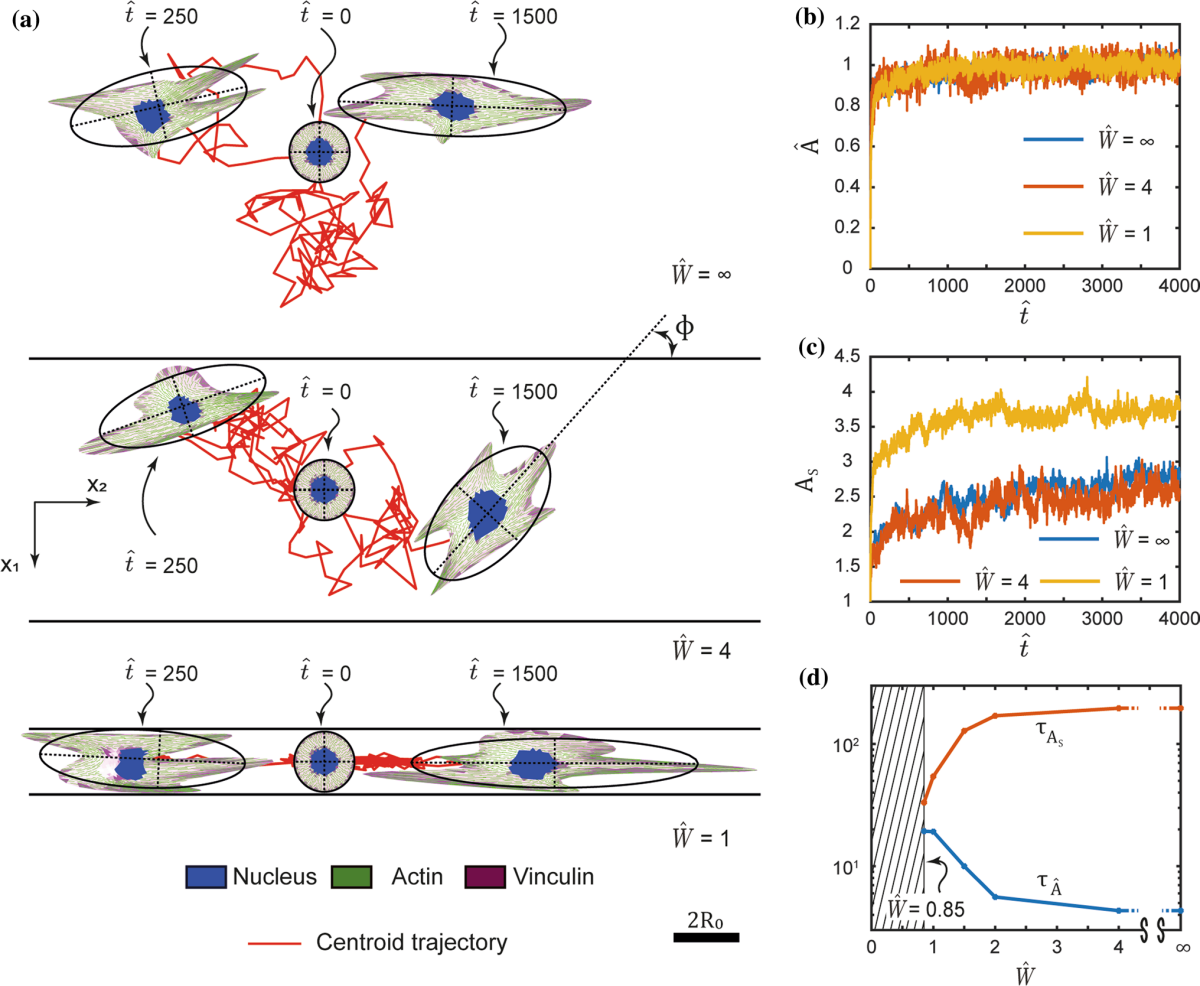
Coupled motility and morphological evolution of the cell seeded on a substrate with adhesive stripes. **a** Two Langevin trajectories for a cell seeded at time $\widehat{t}=0$ from suspension on a a substrate with adhesive stripes of normalised width $\widehat{W}\equiv W/(2{R}_{0})$. One trajectory shows the evolution up to time $\widehat{t}=250$ while the second to $\widehat{t}=1500$. Results are shown for three stripe widths $\widehat{W}=\infty , 4$ and 1. The scalebar $2{R}_{0}$ is the diameter of the circular cell in its elastic rest state **b**, **c**. The corresponding temporal evolution of (b) the normalised cell area $\widehat{A}$ and aspect ratio ${A}_{\mathrm{S}}$ averaged over $n\ge 50$ Langevin trajectories. **d** Dependence of the timescales of the evolution of the area and aspect ratio on $\widehat{W}$. Cells no longer remain attached to the stripes for $\widehat{W}<0.85$ (Buskermolen et al. 2019), and this region is shown shaded

Given a cell seeded in the middle of the stripes at time $\widehat{t}=0$ from suspension, Fig. 9a shows snapshots of the cell morphology and position from a single trajectory on stripes of normalised width $\widehat{W}\equiv W/(2{R}_{0})=4$ and $1$ as well as the unpatterned substrate with $\widehat{W}=\infty$. The snapshots are shown for two selected times along with best fit ellipses and the corresponding orientations $\varphi$ of the cell with respect to the stripe. From the $n\ge 50$ trajectories computed here we have selected in Fig. 9a trajectories where the cell motion backed onto itself between times $\widehat{t}=250$ and 1500 so that the cell positions are well separated, and we can easily illustrate the cell morphologies. While clear visual differences between the cell morphologies are observed for the cell on the different stripes, the most striking differences are in the cell trajectories where we clearly see that the cell is “guided” on the $\widehat{W}=1$ stripe with the trajectory being nearly one-dimensional (also see Supplementary Video 3).

A key outcome of the discussion in Sect. 4 is that on unpatterned substrates the area of the cell evolves much faster than its aspect ratio. Is this feature carried forward to when cells are constrained to within a single adhesive stripe? To investigate this, we observe from Fig. 9b and 9c that the key morphological variables of interest, viz. the area and aspect ratio, evolve in approximately an exponential manner. Thus, we fit a curve of the form

33 $${\text{x}}\left(\widehat{t}\right)={\mathrm{x}}_{\infty }-\left({\text{x}}_{\infty }-{\text{x}}_{R}\right)\mathrm{exp}\left(-\frac{\widehat{t}}{{\tau }_{\text{x}}}\right) ,$$

to their temporal evolution averaged over $n$ trajectories. Here, $\mathrm{x}$ denotes the value of the observable of interest (i.e. either normalised area $\widehat{A}$ or aspect ratio ${A}_{\mathrm{S}}$) with ${\text{x}}_{\infty }$ denoting the value at normalised time $\widehat{t}\to \infty$ while ${\text{x}}_\mathrm{{R}}$ is the value of the observable for the cell in suspension at $\widehat{t}=0.$ While ${\text{x}}_{\infty }$ and ${\text{x}}_\mathrm{{R}}$ are directly available from trajectories as shown in Fig. 9b and 9c, the time constant ${\tau }_{\text{x}}$ is obtained via a best fit of (33) to the Langevin predictions. The inferred time constants $\tau_{{\hat{\mathrm{A}}}}$ and $\tau_\mathrm{{{A_{S} }}}$ for the evolution of the area and aspect ratio, respectively, are included in Fig. 9d as a function of the stripe width $\widehat{W}$. (Cells no longer remain attached to the stripes for $\widehat{W}<0.85$ (Buskermolen et al. 2019), and this region is shown shaded.) With decreasing $\widehat{W}$ the aspect ratio evolves faster while the time for the area to attain its steady-state value increases. Consequently, $\tau_{{\hat{\mathrm{A}}}}$ and ${\tau }_\mathrm{{{A}_{S}}}$ converge towards each other at small $\widehat{W}$ eliminating the difference in the timescales for the area and aspect ratio evolution seen for the unpatterned substrates. For cells seeded on stripes, even constraining ourselves to spatially uniform elliptical cells results in a complex energy landscape as not all values of the $({\lambda }_{1},{\lambda }_{2})$ are permissible for all cell orientations $\varphi$. Thus, a simple picture like that presented in Fig. 7 cannot be presented here to explain the convergence of the timescales for the evolution of area and aspect ratio with decreasing $\widehat{W}$. However, the only physics in the model that sets the timescales is the topology of the energy landscape. Hence, we conclude that the changes in the free-energy landscape due to the confining effect of the narrow stripes guide the area and aspect ratio of the cell to evolve hand-in-hand.

### Timescale for the development of guidance and non-diffusive motility of cells on substrates with adhesive stripes

From the evolution of the morphological observables, we have discussed the dynamic effect of guidance, but we have yet to characterise its properties. This guidance can be characterised in two ways: (i) via the cell orientation that is related to cell morphology and (ii) via the direction of motion. Here, we first focus on cell orientation and in the following section on guidance which is more directly a consequence of motility.

**Fig. 10:**
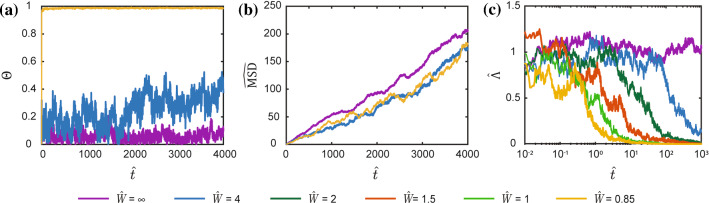
**a** Evolution of the order parameter Θ for different stripe widths. **b, c** Motility of cells on substrates with adhesive stripes. Predictions of the temporal evolution of (**b**) normalised mean square displacement $\widehat{\mathrm{MSD}}$ and **c** dimensionality $\widehat{\Lambda }$ of the motility on stripes of normalised width $\widehat{W}\equiv W/(2{R}_{0})$. The cell is seeded from suspension at time $\widehat{t}=0$ on the stripes

To characterise the cell orientation related to changes in cell morphology with stripe width, we plot in Fig. 10a the order parameter $\Theta$ defined as

34 $$\Theta (\widehat{t})\equiv \sqrt{{\left(\frac{1}{n}{\sum }_{i=1}^{n}\mathrm{cos}2{\varphi }_{(i)}(\widehat{t})\right)}^{2}+{\left(\frac{1}{n}{\sum }_{i=1}^{n}\mathrm{sin}2{\varphi }_{(i)}(\widehat{t})\right)}^{2}},$$

where ${\varphi }_{(i)}$ is the orientation of the cell in the ${i}^{\mathrm{th}}$ trajectory at time $\widehat{t}$ (see Fig. 9a for the definition of ${\varphi }_{(i)}$ as the inclination of the major axis of the best fit ellipse to the stripe direction). The order parameter $\Theta$ is defined such that $\Theta =0$ if ${\varphi }_{(i)}$ is uniformly distributed over all $n$ trajectories, while $\Theta =1$ if ${\varphi }_{(i)}$ takes a unique value. Cell alignment as parameterised through $\Theta$ also increases with decreasing $\widehat{W}$ although the large increases in $\Theta$ occur at around $\widehat{W}\approx 1$ when the aspect ratio increases. The increase in cell alignment at steady state with decreasing $\widehat{W}$ has been reported in (Buskermolen et al. 2019). In that study, the authors argue that alignment results from the fact that cells near the edge of the stripes are necessarily aligned with the stripes: this boundary effect is of course more prominent for narrower stripes and also with cell elongation. Consequently, cells are more aligned for smaller $\widehat{W}$ in line with the HLE predictions. Since alignment is a result of cells sensing the stripe edges, it follows that alignment is a consequence of cells wandering over the stripes including towards the edge of the stripes and hence an outcome of cell motility. Thus, not only does $\Theta$ increase with decreasing $\widehat{W}$ but also the time required for the steady-state value of $\Theta$ to be attained decreases with decreasing $\widehat{W}$. This timescale for the evolution of $\Theta$ is of course set by the motility timescales of cells on the stripes.

To quantify the motility of cell on stripes, we include predictions of the variation of $\widehat{\mathrm{MSD}}$ ($n\ge 50)$ with $\widehat{t}$ on stripes of different widths in Fig. 10b. Unlike the case of the unpatterned substrate ($\widehat{W}=\infty$), the $\widehat{\mathrm{MSD}}$ on stripes of width similar to cell size (i.e. the $\widehat{W}=0.85$ and 4 cases shown in Fig. 10b) does not seem to vary linearly with $\widehat{t}$. To understand the effect of the constraint of the stripes on motility, we introduce a measure of the mean square displacement in the direction of the stripes, viz.

35 $${\widehat{\mathrm{MSD}}}_{y}=\frac{1}{n{R}_{0}^{2}}{\sum }_{i=1}^{n}{y}_{\left(i\right)}^{2}(t),$$

where the seeding location at time $t=0$ is assumed to be the origin of the co-ordinate system ($y\equiv {x}_{2}-$ aligned with the stripe direction as shown in Fig. 9a). To parameterise the influence of the constraint of the stripes, we then define

36 $$\widehat{\Lambda }\equiv 2\frac{\widehat{\mathrm{MSD}}-{\widehat{\mathrm{MSD}}}_{y}}{\widehat{\mathrm{MSD}} }.$$

This parameter that characterises the dimensionality of the motility is defined such that $\widehat{\Lambda }=1$ on an unpatterned substrate as $\widehat{\mathrm{MSD}}=2{\widehat{\mathrm{MSD}}}_{y}$, since movements in the $x\equiv {x}_{1}$ and $y-$ directions are free and non-distinguishable, while $\widehat{\Lambda }\to 0$ for $\widehat{W}\ll 1$ when the motion of the cell is completely constrained to be only in the $y-$ direction (i.e. is one-dimensional) so that $\widehat{\mathrm{MSD}}={\widehat{\mathrm{MSD}}}_{y}$. Predictions of the temporal variation of $\widehat{\Lambda }$ are included in Fig. 10c for selected stripe widths $\widehat{W}$. In all cases, $\widehat{\Lambda }\approx 1$ at early stages of the cell motion, but, except for the unpatterned substrate ($\widehat{W}=\infty )$, $\widehat{\Lambda }$ subsequently reduces to 0. However, the time at which this transition from 2 to 1D motility occurs increases with increasing $\widehat{W}$. This can be understood by recognising that the $x-$ displacement of the cell is constrained by the stripe width while the $y-$ direction displacement is unconstrained and thus with increasing time $\widehat{\mathrm{MSD}}$ is dominated by the $y-$ direction displacement and $\widehat{\mathrm{MSD}}\to {\widehat{\mathrm{MSD}}}_{y}$. The transition from a two-dimensional (2D) motion of the cell during the early stages of cell motion to 1D motility in the later stages (with the transition time being $\widehat{W}$ dependent) induces the loss of the linear dependence of $\widehat{\mathrm{MSD}}$ with time seen in Fig. 10b for the finite stripe widths. Moreover, we would also anticipate that the attainment of the steady state of the order parameter $\Theta$ is governed by the time of transition from 2D to 1D motility. A comparison of Fig. 10a with 10c confirms that indeed $\Theta$ attains it steady-state value at approximately the time that $\widehat{\Lambda }\to 0$ and thus the time to achieve steady-state alignment decreases with decreasing $\widehat{W}$. Finally, we note that the transition of $\widehat{\Lambda }$ from 1 to 0 denotes the guidance of the cells by the stripes with the time taken for $\widehat{\Lambda }$ to transition from 1 to 0 the time for contact guidance to be achieved on the patterned substrate.

### The dynamics of biochemical alignment are strongly dependent on the morphological evolution

So far we have discussed the effect of confinement on cell motility and evolution of morphological observables. The model also explicitly includes the stress-fibre polymerisation that are strongly linked to cell morphology and here present predictions of the HLE for the evolution of the cytoskeletal stress-fibre arrangements in terms of a cytoskeletal order parameter that we now proceed to define.

Given the spatial distribution of the direction ${\phi }_{\mathrm{max}}$ of the dominant stress-fibre bundle (set by the maximum value of $\widehat{\eta } {\widehat{n}}^{\mathrm{ss}})$ at any location ${x}_{i}$, we then define a rotationally invariant spatial distribution of stress-fibre orientations as

37 $$\widehat{\phi }\equiv {\phi }_{\mathrm{max} }-\frac{1}{{V}_{\mathrm{C}}}{\int }_{{V}_{\mathrm{C}}}{\phi }_{\mathrm{max} } dV .$$

**Fig. 11:**
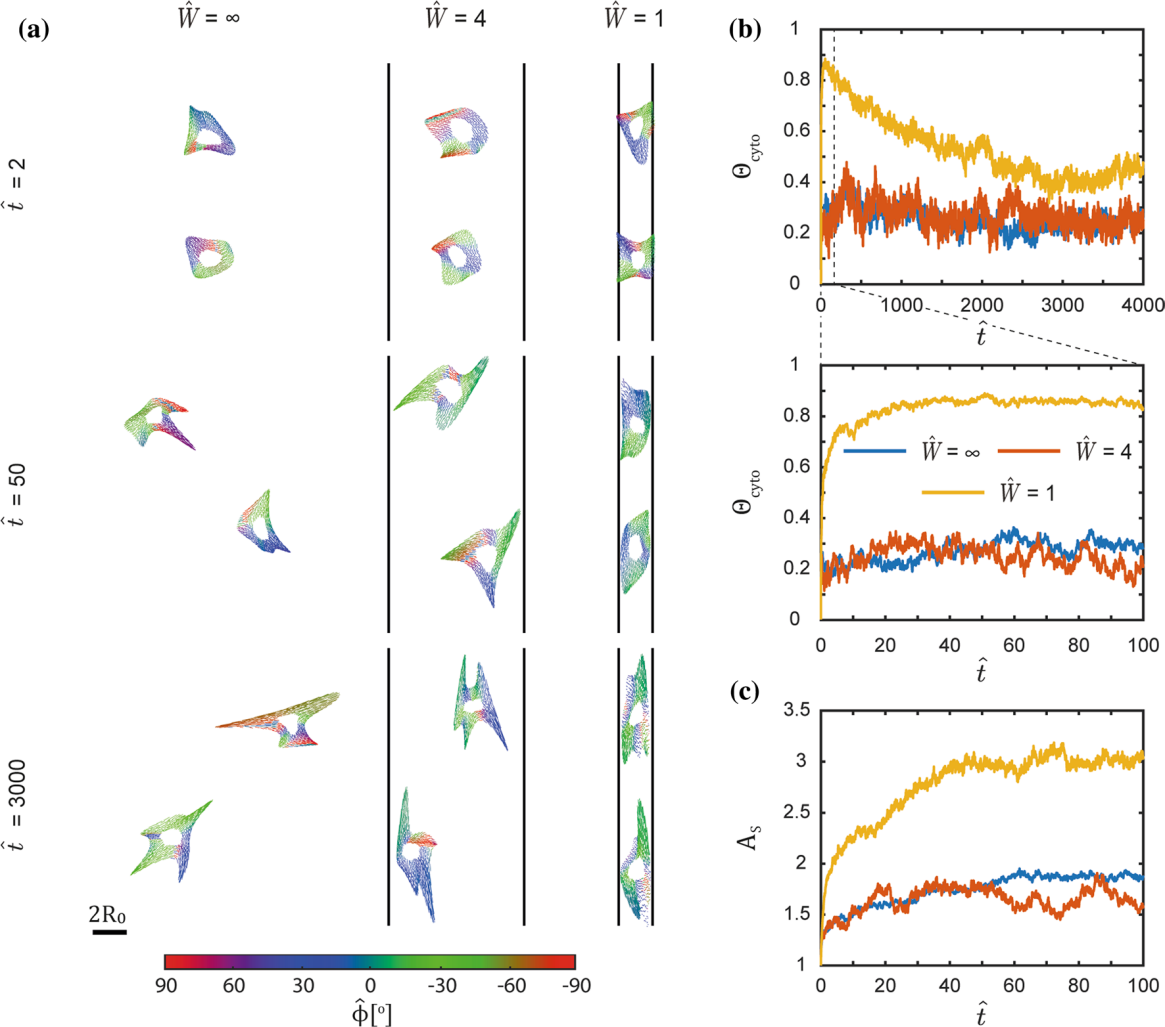
**a** Snapshots of the distribution of stress-fibre orientation $\widehat{\phi }$ for cells seeded on stripes of different normalised widths $\widehat{W}$ at selected times $\widehat{t}$. **b** Evolution of the cytoskeletal order ${\Theta }_{\mathrm{cyto}}$ as a function of $\widehat{t}$ with a zoom-in showing the early time evolution. **c** Evolution of the aspect ${A}_{S}$ in the early stages corresponding to the zoom-in in (**b**). The cell is seeded from suspension at time $\widehat{t}=0$ on the stripes

Predictions of the spatial distribution of $\widehat{\phi }({x}_{i})$ for cells seeded on stripes of different widths and at selected times $\widehat{t}$ in a given HLE trajectory are shown in Fig. 11a (also see Supplementary Video 4). Homogeneity of colour in Fig. 11a is an indication of stress-fibre alignment and while the results in Fig. 11a suggest that stress-fibre alignment increases with time, the more dominant effect is seen with decreasing stripe width. To quantify this, we define a stress-fibre order parameter ${\Theta }_{\mathrm{cyto}}$ analogously to (34) with $\varphi$ replaced by $\widehat{\phi }$. The temporal evolution of ${\Theta }_{\mathrm{cyto}}$ for $\widehat{W}=\infty , 4, 1$ (averaged over $n\ge 50$ HLE trajectories) is shown in Fig. 11b. While the predictions for $\widehat{W}=\infty$ and 4 are nearly identical, a rather interesting feature is observed for the $\widehat{W}=1$ case with ${\Theta }_{\mathrm{cyto}}$ increasing rapidly and then decaying to a steady-state value that is higher than that for $\widehat{W}=\infty$*.* To better understand this rapid increase in ${\Theta }_{\mathrm{cyto}}$, we show in Fig. 11b a zoom-in for $\widehat{t}\le 100$ where we observe that the maximum value of ${\Theta }_{\mathrm{cyto}}$ is attained at $\widehat{t}\approx 50$. To examine the relevance of this time, we include in Fig. 11c the corresponding temporal evolution of the aspect ratio where we observe a biphasic evolution: a rapid increase for $\widehat{t}\le 50$ followed by a slower increase. We hypothesise that the rapid increase in aspect ratio is accompanied by or even requires high stress-fibre alignment and subsequently the alignment relaxes to increase the entropy of the stress-fibres and decrease cell free energy. These complex couplings are natural outcomes of the HLE.

## Concluding discussion

The temporal response of isolated cells on unpatterned and patterned substrates has been investigated via a novel framework, labelled the homeostatic Langevin equation (HLE), that recognises the non-thermal fluctuations of cells. The morphological evolution is driven by gradients of the cell free energy, and we show that the HLE correctly predicts that the cell area or spreading evolves at a rate an order of magnitude faster than cell aspect ratio or elongation. These two very different timescales emerge as a consequence of the interplay between the stress-fibre polymerisation and cell passive elasticity. The framework enables the prediction of the coupled evolution of cell morphology along with cell motility. Over the timescales when the HLE is applicable, the simulated cell motility on an unpatterned substrate is Brownian, in line with numerous observations (Stokes et al. 1991; Plou et al. 2018; Dunn and Brown 1987), and emerges from coordinated morphological fluctuations.

On substrates patterned with adhesive stripes cells again spread and elongate much like on unpatterned substrates. However, key differences now emerge related to the fact that the Brownian motion of the cells is now restricted outside of the parallel direction to the stripes. For example, for cells seeded on stripes the timescales for the evolution of area and aspect ratio evolution converge with decreasing width of the stripes. Furthermore, for narrow stripes, the evolution of the stress-fibre cytoskeletal arrangement shows a biphasic behaviour with an initial rapid rise associated with a rapid increase in the cell aspect ratio and then a decay to its steady-state value. The differences in the dynamics of the morphological observables carry to cell motility too. The HLE predicts that if sufficient time is given for the cells to explore the stripe widths, their 2D Brownian motion reduces to one-dimensional (1D) motion along the length of the stripes. This results in an apparent non-Brownian effect with the mean-square-displacement of the cell centroid no longer scaling linearly with time over the entire duration. A more important consequence of this switch to 1D motility is contact guidance or rather 1D motion of the cell along the stripes which manifests itself also in terms of the alignment of the cell orientation with the stripe direction. Thus, a key conclusion of the HLE framework is that the non-thermal cell fluctuations give the cell its ability to explore the stripe width and in turn, rather counterintuitively, result in its guidance and alignment with the anisotropy of its environment.

### Electronic supplementary material

Below is the link to the electronic supplementary material.

Supplementary file1 (DOCX 13 kb)

Supplementary file2 (MP4 3568 kb)

Supplementary file3 (MP4 12022 kb)

Supplementary file4 (MP4 18225 kb)

Supplementary file5 (MP4 6318 kb)

## Sampling of the homeostatic ensemble

The probability distribution (1) is sampled by constructing a Markov chain that serves as a sample of the homeostatic ensemble for cells on substrates. The algorithm closely follows the approach developed by Shishvan et al. (2018) and Buskermolen et al. (2019) and uses the Metropolis (1953) algorithm in an iterative manner using the procedure explained in detail in (Shishvan et al. 2018). From the algorithm, the normalised homeostatic temperature is defined as $1/\widehat{\zeta }$ $\equiv 1/\zeta |{G}_{\mathrm{S}}|$, where $\zeta$ is the homeostatic temperature and ${G}_{\mathrm{S}},$ the Gibbs free energy of the cell in suspended state, is obtained. An outline of the Markov chain Monte Carlo scheme using Metropolis sampling (Metropolis 1953) is as follows:

(i) Assume a value of $\zeta$ and perturb the undeformed cell configuration to obtain an initial random configuration labelled as the morphological microstate at $j=0$ and calculate the Gibbs free energy ${G}^{(j=0)}$ of that state.
(ii) Randomly pick a control point $\left[{}^{(q)}{r}_{1},{}^{(q)}{r}_{2}\right]$ and perturb both degrees of freedom by random values picked from a uniform distribution over the interval $\left[-\Delta ,\Delta \right]$.
(iii) Compute the new free energy ${G}^{(j)}$ of this perturbed state and the change in free energy $\Delta G={G}^{(j)}-{G}^{(j-1)}$.
(iv) Use the Metropolis criterion to accept the perturbed state or not, i.e.
  - $\Delta G\le 0$, accept the perturbed state.
  - for $\Delta G>0$, compute ${P}^{\mathrm{acc}}=\mathrm{exp}\left(-\zeta \Delta G\right)$ and accept the perturbed state if ${P}^{\mathrm{acc}}>\mathcal{P}$, where $\mathcal{P}$ is a random number sampled from the uniform distribution $[0 1]$.
(v) If the perturbed state is accepted, add it to the list of samples as a new morphological microstate else repeat the configuration $(j-1)$ in the sample list. Return to step (ii).
(vi) Keep repeating this procedure until a converged distribution is obtained. Typical Markov Chains consist of $\mathcal{N}=1$ million samples.
(vii) After running $k$ different Markov Chains (to ensure that the correct sampling of the phase space), calculate the ensemble Gibbs free energy $\langle {G}^{(j)}\rangle =\frac{1}{N}\sum_{j=1}^{N}{G}^{(j)}$, where $N=k\mathcal{N}$ is the total number of sampled configurations over the $k$ Markov Chains. If $\langle {G}^{(j)}\rangle$ is within $\pm 2\%$ of ${G}_{\mathrm{S}}$, then this distribution is accepted, else the value of $\zeta$ is modified and the process is repeated from (i).

A “burn-in” number of initial microstates from each Markov chain are discarded in the chain to eliminate the dependency of the sampled configurations from the initial random guess. The value of burn-in we used is $\mathrm{50,000}$. As is typical in such Markov chain Monte Carlo (MCMC) calculations, we attempted to achieve an acceptance rate of about 35% in the Metropolis criterion and adjusted $\Delta$ to ensure that we stayed within $\pm 5\%$ of this target acceptance rate. All algorithms, including the free energy evaluations, were run using in-house developed Matlab codes, and the duration to compute a typical Markov chain is between 1 and 2 h.
