# Supplementary material for: Contact guidance as a consequence of coupled morphological evolution and motility of adherent cells
Source: Biomech Model Mechanobiol. 2022 Apr 27;21(4):1043–65. doi: 10.1007/s10237-022-01570-9 (PMC9283373; doi:10.1007/s10237-022-01570-9)
Supplement: Supplementary file 1 — Supplementary file1 (DOCX 13 kb) [file 10237_2022_1570_MOESM1_ESM.docx]

**Supplementary Video captions**

**Supplementary Video 1:** Morphological evolution of a cell. The image of the cell shows the evolution of a cell over a single simulated HLE trajectory after seeding from suspension at normalized time $\hat{t}=0$. The corresponding evolution of the ensembled averaged morphological observables (normalized area $\hat{A}$, aspect ratio $A_{s}$ and form factor $FF$) is also included along with an indicator showing the time corresponding to the image of the cell in the movie.

**Supplementary Video 2:** Motility of a cell. The image of the cell shows the coupled motility and morphological evolution of a cell over a single simulation HLE trajectory after seeding from suspension at normalized time $\hat{t}=0$. The corresponding temporal evolution of the normalized squared displacement $\hat{\mathcal{R}}(\hat{t})$ is also included along with an indicator showing the time corresponding to the image of the cell in the movie. The red line shows the path of the centroid of the cell.

**Supplementary Video 3:** Contact guidance of cells. The images of the cells show the coupled motility and morphological evolution of the cells over a single simulation HLE trajectory after seeding from suspension at normalized time $\hat{t}=0$ on stripes of two different widths $W$ in addition to the 2D substrate where $W=\infty$. The red line shows the path of the centroids of each cell.

**Supplementary Video 4:** Stress-fibre ordering for cells seeded on stripes. The images show the cell morphology and cytoskeletal arrangements evolving over a single simulation HLE trajectory after seeding from suspension at normalized time $\hat{t}=0$ on stripes of two different widths $W$ in addition to the 2D substrate where $W=\infty$. The stress-fibres are coloured by the orientation $\hat{\phi}$ in the manner done in Fig. 11a. The corresponding predictions of $\Theta_{\mathrm{cyto}}$ are also included.
